# Chromatographic reversed HPLC and TLC-densitometry methods for simultaneous determination of serdexmethylphenidate and dexmethylphenidate in presence of their degradation products—with computational assessment

**DOI:** 10.1186/s13065-023-00986-3

**Published:** 2023-07-15

**Authors:** Khadiga M. Kelani, Ahmed M. W. Nassar, Gamal A. Omran, Samir Morshedy, Ahmed Elsonbaty, Wael Talaat

**Affiliations:** 1 Pharmaceutical Analytical Chemistry Department, Faculty of Pharmacy, Cairo University, Kasr El-Aini Street, Cairo, ET- 11562 Egypt; 2 Pharmaceutical Analytical Chemistry Department, Faculty of Pharmacy, Modern University for Technology and Information (MTI), Cairo, Egypt; 3 Pharmaceutical Analytical Chemistry Department, Faculty of Pharmacy, Damanhour University, Damanhour, Egypt; 4 Pharmaceutical Chemistry Department, Faculty of Pharmacy, Egyptian Russian University, Badr, 11829 Cairo Egypt

**Keywords:** Serdexmethylphenidate (SER.DMP), Dexmethylphenidate (DMP), HPLC–DAD, TLC-densitometry, SER.DMP and DMP degradation products, Molecular dynamic simulation

## Abstract

Two Chromatographic methods have been established and optimized for simultaneous determination of serdexmethylphenidate (SER.DMP) and dexmethylphenidate (DMP) in the presence of their degradation products. The first method is a reversed phase high performance liquid chromatography with diode array detection (HPLC–DAD). Isocratic separation was carried out on Waters X-bridge Shield RP_18_ column (150×3.9×5 μm particle size) using a mixture of 5 mM phosphate buffer (pH 5.5): acetonitrile (40:60, v/v) as a mobile phase, flow rate 1 mL/min and detection at 220 nm. The second method is a thin-layer chromatography (TLC)—densitometry method using methanol: chloroform (70:30, v/v) as a mobile phase and UV scanning at 220 nm. In HPLC method, the linearity range of SER.DMP was (2.5–25 μg/mL); with LOD (0.051 μg/mL) and LOQ (0.165 μg/mL) while for DMP was (2.5–25 μg/mL); with LOD and LOQ of (0.098 μg/mL) and (0.186 μg/mL), respectively. For TLC method the sensitivity range of SER.DMP was (5–25 μg/mL), LOD was (0.184 μg/spot), while LOQ was (0.202 μg/ spot) whereas for DMP the sensitivity range was (5–25 μg/mL) with LOD of (0.115 μg/ spot) and LOQ of (0.237 μg/ spot), respectively. SER.DMP was found to be equally labile to acidic and alkaline hydrolysis, whereas DMP was sensitive to acidic hydrolysis only. Both drugs were successfully determined in presence of acidic and basic degradants by the two developed methods (stability indicating assay method). Chromatographic separation of the degradation products was carried out on TLC aluminum silica plates 60 F254, as a stationary phase, using methanol: dichloroethane: acetonitrile (60:20:20 v/v), as a mobile phase. The degradation pathway was confirmed using TLC, IR, ^1^H-NMR and mass spectroscopy; moreover, the separation power was correlated to the computational results by applying molecular dynamic simulation. The developed methods were validated according to the International Conference on Harmonization (ICH) guidelines demonstrating good accuracy and precision. They were successfully applied for quantitation of SER.DMP and DMP in pure and capsule forms. The results were statistically compared with those obtained by the reported method in terms of accuracy, precision and robustness, and no significant difference was found.

**Supplementary Information:**

The online version contains supplementary material available at 10.1186/s13065-023-00986-3.

## Introduction

**Fig. 1:**
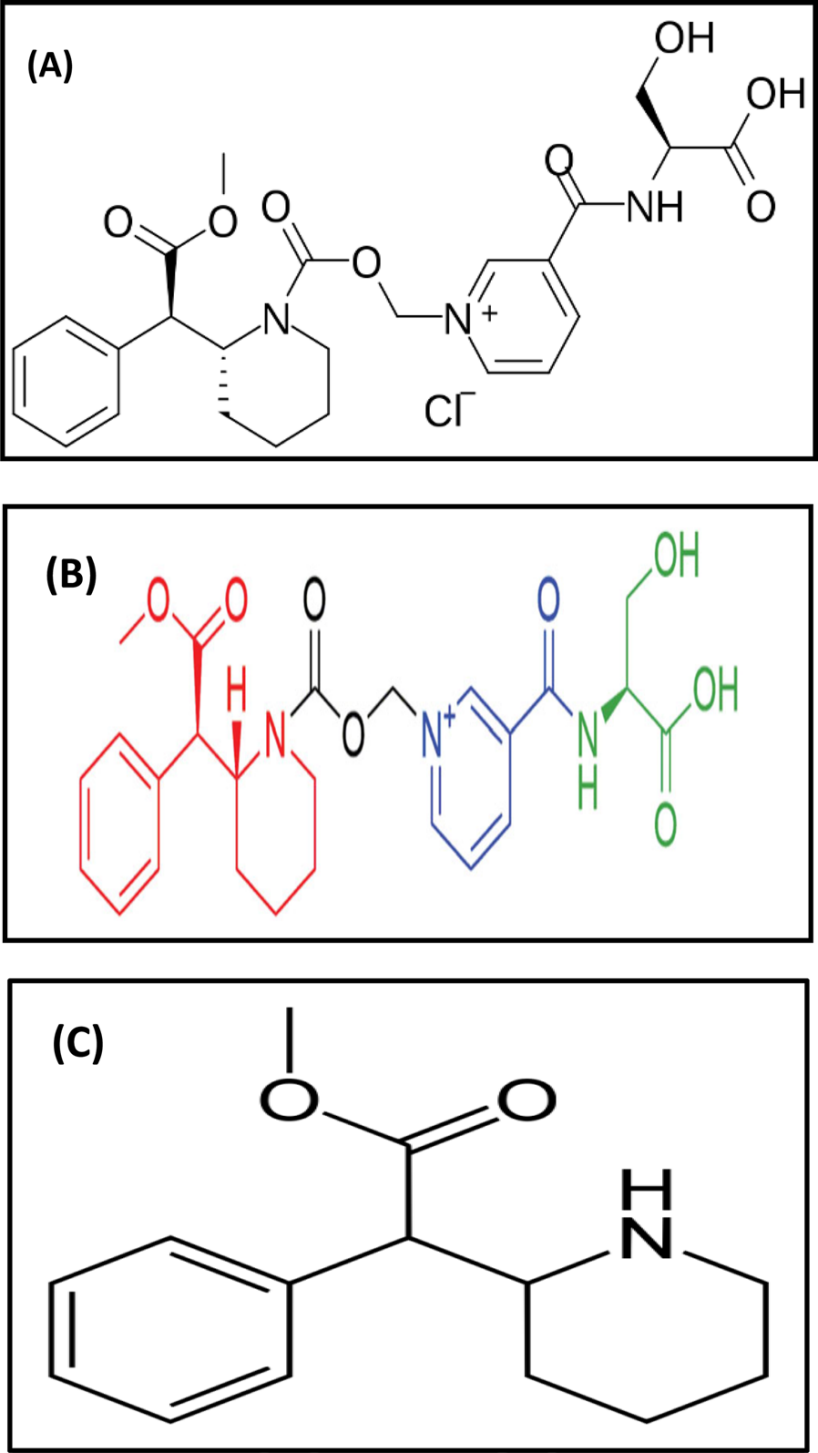
**A**: Structural formula of Serdexmethylphenidate chloride (SER.DMP) **B**: Structural formula of molecular components of Serdexmethylphenidate **C**: Structural formula of Dexmethylphenidate (DMP)

Serdexmethylphenidate (SER.DMP) and Dexmethylphenidate (DMP) belong to the group of medicines called central nervous system (CNS) stimulants [1, 2]. Both drugs are present together in the form of tablets and capsules for oral use. Their combination is used for the treatment of Attention Deficit Hyperactivity Disorder (ADHD) in patients 6 years of age and older [1]. SER.DMP is a prodrug of DMP and it is pharmacologically inactive until gradually converted to active DMP in the lower gastrointestinal tract [3]. As a prodrug, SER.DMP has a delayed onset of action and a prolonged duration of effects compared to DMP. Thus, co-formulation of SER.DMP with DMP allows for a more rapid onset of action while still retaining longer therapeutic efficacy [3]. In addition, SER.DMP has lower abuse potential than DMP [4]. Dexmethylphenidate (DMP) appears to block the reuptake of norepinephrine and dopamine into the presynaptic neuron, increasing their availability in the extracellular space. However, the mechanism of action in Attention Deficit Hyperactivity Disorder is unknown. SER.DMP / DMP was granted FDA approval in March 2021, and is currently marketed as a fixed-dose combination drug under the tradename Azstarys^®^. This combination of the drugs allows Azstarys to work throughout the day. The chemical name of SER.DMP chloride is (2S)-3-hydroxy-2-[[1-[[(2R)-2-[(1R)-2-methoxy-2-oxo-1-phenylethyl] piperidine-1-carbonyl] ox methyl] pyridin-1-ium-3-carbonyl] amino] chloride propionic acid [1, 2]; its structural formula is shown in Fig. 1A. It consists of a single d- DMP molecule covalently attached via a carbamate bond to a methylene oxide linker, which in turn is connected to a nicotinoyl-serine moiety, as illustrated in Fig. 1B [4]. The chemical name of DMP is (R,R)-( +)-Methyl 2-phenyl-2-(2-piperidyl)acetate [1, 2], {the d-( +) throe-enantiomer of methylphenidate} [1, 2] and its structural formula is shown in Fig. 1C. A literature review revealed that both drugs were simultaneously determined using HPLC methods [5–7]. None of the reported methods identified the degradation products. The aim of this work was to develop stability indicating chromatographic methods for the determination of both drugs in presence of their acidic and alkaline degradation products using Diode Array Detector (DAD) for detection of all possible degradation products through multiple monitoring processes, chromatographic separation and identification of degradation products by TLC, IR,NMR and mass spectroscopy, and application of Computational Correlation Assessment [8] to illustrate the efficacy of HPLC column separation [8].

## Experimental

### Instruments

HPLC was carried on LDC Analytical Waters, USA, equipped with Diode-array UV–Visible detector and auto sampler injector. The chromatographic analysis was carried out using EZ Chrome Elite data analysis program and Waters X-bridge Shield RP_18_ column [(150 × 3.9 × 5 μm particle size) B.N: WAT046980, (S.N:0200343221)]. Camag Micro syringe (100 μL) Lino mat autosampler is from Camag^®^ company, Automatic TLC Sampler 4 (ATS 4), Muttenzl, Switzerland. Camag TLC scanner (35/N/30319) with win CATS software; an ultraviolet (UV) lamp with a short wavelength at 250 nm (Desaga, Wiesloch, Germany). TLC analysis was carried on Aluminum TLC plates precoated with silica gel 60 GF_254_ (20 × 20 cm, Merck, Darmstadt, Germany) with its chromatographic tank (25 × 25 × 9 cm). Other equipment used: hot plate (Torrey pines Scientific, USA); Jenway, 3510 pH meter (Jenway, USA); Scilogex Rotatory evaporator; NMR, Gemini-400 BB (Agilent, USA); FT-IR, Nicolet IR 200 (Thermo electron corporation, USA), with MS-QP-1000 EX mass spectrometer at 70 eV (Shimadzu, Tokyo, Japan); molecular operating environments (MOE) {2015:10} software (Chemical Computing Group, Montreal, QC, Canada) were used.

### Materials and reagents

Serdexmethylphenidate (99.55 % purity) and dexmethylphenidate (99.70 % purity) were kindly provided by Novartis Pharma S.A.E., Cairo, Egypt. Azstarys capsules (batch number R-11052) manufactured by Novartis Pharma S.A.E., Cairo, Egypt, under license from Novartis Consumer Health SA—Switzerland; labeled to contain 52.3 mg of serdexmethylphenidate and 10.4 mg dexmethylphenidate per capsule. Acetonitrile, dichloroethane and methanol, all of HPLC grade (Sigma-Aldrich, Germany). Hydrochloric acid and Sodium hydroxide, analytical grade (El-Nasr Co., Egypt), were prepared as 0.1N aqueous solutions. Phosphate buffer (pH 5.5) was prepared.

### Standard solutions

For the preparation of SER.DMP and DMP (1 mg/mL) stock solutions, 0.1 gm (equal to 100 mg) of pure SER.DMP and DMP were separately dissolved in 50 mL of methanol and the volume completed to 100 mL with methanol. Working solutions at various concentrations (varying from 2.5 to 25 μg/mL) were prepared by appropriate dilution of the stock solution.

### Procedures

#### A) Acidic and basic degradation of SER.DMP and DMP

Acidic and basic hydrolysis were carried out using 25 mg of each drug and 50 mL of 0.1 N HCl, or 0.1 N NaOH and reflux for 2 h at 60 °C. Complete degradation was verified by TLC plate using methanol: chloroform (70:30 v/v) as a mobile phase. Each sample was then neutralized with 0.1N alkali or 0.1N acid, evaporated to dryness, and dissolved by 50 mL of methanol, transferred into 100 mL volumetric flask, and completed to the mark with methanol to obtain a stock concentration equivalent to 250 μg/mL of each drug degradant [9–11].

#### B) Isolation and identification of degradants

Preparative TLC using aluminum silica plates GF_254_ as stationary phase, and methanol:dichloroethane: acetonitrile (60:20:20 v/v) as mobile phase was used for the separation of degradants. After complete separation, each degradant was scratched and extracted twice with methanol and dried at 25–30 °C to obtain pure solid form. Isolated degradants were subsequently subjected to infrared/nuclear magnetic resonance/mass spectral analyses for identification [10, 11].

#### Construction of calibration curves

##### Reversed phase-HPLC method

Isocratic separation was carried out on Waters X-bridge Shield RP_18_ column (150 × 3.9 × 5 μm particle size) at column temperature 25 °C, using mobile phase consisting of a mixture of 5 mM phosphate buffer (pH 5.5): acetonitrile (40:60 v/v)]. The mobile phase was degassed by a degasser before being pumped at flow rate 1 mL/min and detection at 220 nm. Aliquots of standard SER.DMP solution (1 mg/mL) equivalent to 2.5—25 μg were transferred into a series of 10-mL volumetric flasks and adjusted to volume with the mobile phase. The same was carried out for DMP. A 20 µL from each solution was injected into HPLC column and eluted with the mobile phase under the previously described chromatographic conditions. Calibration curves representing the relationship between peak area and the corresponding concentration μg/mL of SER.DMP and DMP were plotted, and the regression equations were computerized.

##### Thin layer chromatography-densitometric method

Aliquots of SER.DMP and DMP stock standard solutions (1 mg/mL) equivalent to (5–25 μg/spot) were transferred to a set of 10-mL volumetric flasks and the volume was then completed to the mark with methanol. A 10 μL of each solution were applied to TLC plate (10 cm × 10 cm) using Camag Lino mat auto sampler with micro syringe (100 μL). The plate was then developed by the ascending technique using methanol: chloroform (70:30 v/v) as a mobile phase. The plate was then removed and air-dried. The chromatogram was scanned at 220 nm. Calibration curves representing the relationship between integrated peak area and the corresponding concentration in μg/spot of SER.DMP and DMP were plotted.

#### Application of the proposed methods to laboratory prepared mixtures

Laboratory prepared mixtures of the intact drugs and degradants were prepared by accurately measuring variable volumes of the stock standard solution (to give a concentration range of 2.5–25 µg/mL) together with aliquots of standard degradants solution. They were transferred into 10 mL volumetric flasks, completed to the mark with the mobile phase in case of HPLC or methanol in case of TLC procedures to form different ratios of the basic and acidic degradation products, then analyzed as described above. Drug concentrations were calculated using the regression equations.

#### Application of the proposed methods to pharmaceutical formulation

Ten capsules of Azstarys ^®^ were emptied and well mixed. An accurately weight amount equivalent to 100 mg of the drug was transferred into 100-mL beaker, 20 mL methanol was added, and the solution was shaken vigorously for 15 min then sonicated for 30 min and filtered into 100 mL volumetric flask; this was repeated twice. The volume was completed to the mark with methanol to obtain a concentration of 1 mg/mL. The general procedures (HPLC and TLC) were repeated using aliquots covering the working concentration ranges.

### Molecular dynamic simulation

The molecular structure of Serdexmethylphenidate was created by using the atoms and bonds of the MOE program's molecular builder function software. The degradation products: dexmethylphenidate, (R-2-(R-2-methoxy-2-oxo-1-phenylethyl) piperidine-1-Carboxylic acid), (S-3-(1-Carboxy-2-hydroxyethyl-carbamoyl) 1-methyl pyridinium chloride, 3-Carboxy-(R-1,2-methoxy-2-oxo-1-phenylethyl) piperidine-1-Carbonyl-oxymethyl-pyridinium, and L-Serine were built up using Chem. Draw professional, then pasted in MOE program, where the protonation was achieved at pH 5.5. The X-bridge shield RP18 column structures were prepared according to Water Company using molecular builder function software. The partial charges were calculated, and the energy was diminished using a proper force field named the Merck Molecular Force-Field (Amber10: EHT). Also, the Born solvation model was selected where the dielectric constants (DEC) of the solvents were taken into consideration and adjusted at 51, which was equivalent to the ratios of the mobile phase used in the chromatographic separations of the introduced drugs. Molecular dynamics simulation was achieved using the Molecular Operating Environment 2015 software. An appropriate field of force named MMFF94x was used, where the molecular dynamic simulations relied on the Nosé-Poincaré-Andersen (NPA) equations of motion was conducted, and the position, velocity, and acceleration were saved after a sample time of 0.5 picoseconds at 290 K. The distance-dependent dielectric model was used for the definite calculations for solvation energy [8].

## Results and discussion

### Development and optimization of the methods

In this work, reversed phase HPLC and TLC-densitometry methods have been developed for the simultaneous determination of SER.DMP and DMP in capsules and in presence of their acidic and basic degradants. A forced acid and alkaline degradation on SER.DMP and DMP was performed to study degradation behavior of the drugs under this forced conditions. High-performance column liquid chromatographic (HPLC) and TLC densitometry methods were validated according to ICH Guide line. The degradants were identified by IR, H-NMR, and mass spectroscopy. The HPLC results were found to be correlated to the computational ones; this illustrates the efficacy of the column chosen to separate the drug and the degradants in order to match the binding energy of the separated drugs.

### Chromatographic conditions

#### RP-HPLC method

Different chrmatographic conditions affecting the chromatographic separation were optimized. Several mobile phases like {(ammonium acetate solution with OPA (orthophosphoric acid) with acetonitrile 50:50 v/v) and (acetonitrile: methanol: 0.1% formic acid (45:45:10 v/v)} were tried in order to separate the mixture of Serdexmethylphenidate (SER.DMP) and dexmethylphenidate (DMP) in different ratios. However, separation of intact (SER.DMP) and (DMP) from their degradation products were not successful. Good separation was carried out on Waters X-bridge Shield RP_18_ column [(150 × 3.9 × 5 μm particle size)] using mobile phase consists of [5 mM phosphate buffer; pH 5.5: acetonitrile (40:60 v/v) at flow rate (1 mL/min) and UV detection at 220 nm]. The acidic and basic degradation studies were carried out on SER.DMP by first evaluating their degradation behaviour by monitoring the chromatograms of SER.DMP degradation products using the diode array detector to detect any degradation present in the samples and checking the purity of the eluted peaks. Then, acidic degradation study was carried out on DMP to evaluate its degradation behaviour by monitoring the chromatograms of DMP degradation product at 220 nm.

Additional file 1: Fig. S1 shows a typical chromatogram of SER.DMP with retention time of (7.75 ± 0.062 min). For more confirmation of the separation process, the UV absorption curve of serdexmethylphenidate was recorded and it showed typical matching with the curve extracted from the DAD (Additional file 1: Figs. S1 and S2). Study of HPLC chromatograms of SER.DMP and DMP indicated loss of drugs and formation of a variety of degradation products under acid and alkaline forced decomposition conditions. Based on the results obtained, the degradation pathways of SER.DMP and DMP under the prescribed forced degradation conditions are suggested. Isolated degradants were subsequently subjected to infrared/nuclear magnetic resonance/mass spectral analyses studies for identification. Molecular Dynamic Simulation Technique [8] was also applied.

**Fig. 2:**
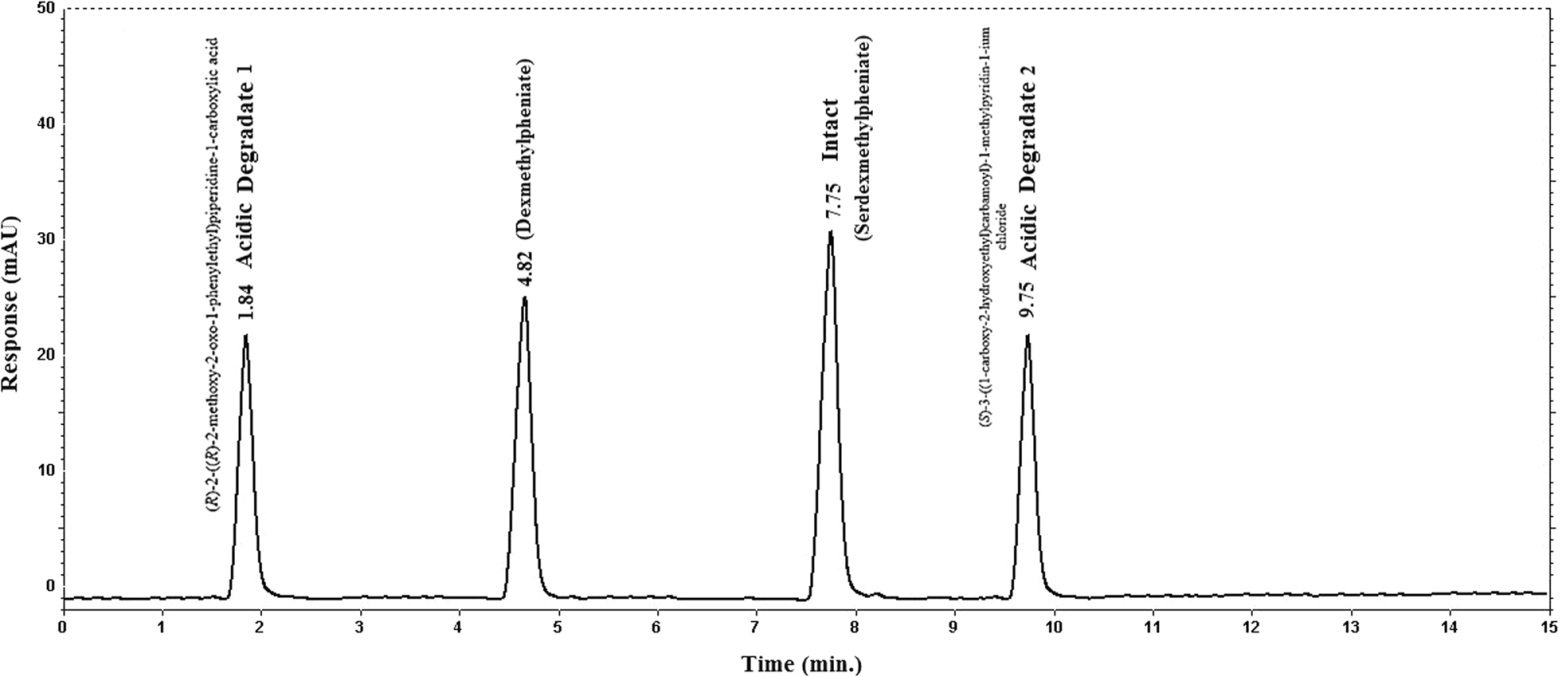
HPLC chromatogram of intact SER.DMP (10 µg/mL) and its acidic degradates

**Fig. 3:**
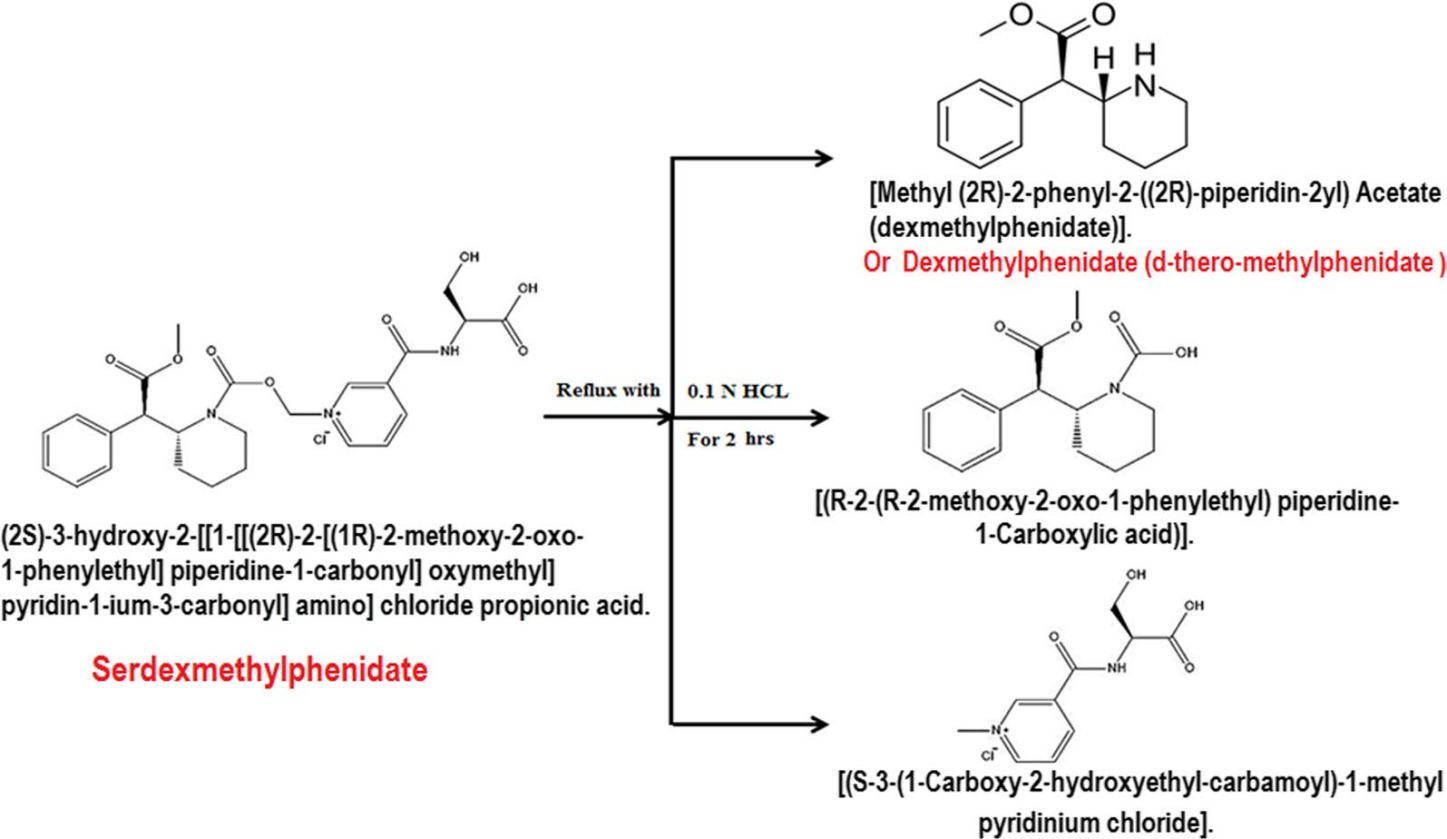
Suggested acidic degradation pathway of SER.DMP

The acidic degradation of pure SER.DMP resulted in three acidic degradants compounds (Fig. 2): (A) [(R-2-(R-2-methoxy-2-oxo-1-phenylethyl) piperidine-1-Carboxylic acid)], (B) [(S-3-(1-Carboxy-2-hydroxyethyl-carbamoyl) -1-methyl pyridinium chloride] and (C) [Methyl (2R)-2-phenyl-2-((2R)-piperidin-2yl) acetate (dexmethylphenidate)]. Suggested acidic degradation pathway of SER.DMP is presented in Fig. 3. HPLC chromatogram revealed that (SER.DMP) and (DMP) were clearly separated from their acidic degradation products at retention times of (4.8 + 0.02 and 7.7 + 0.05) for (DMP) and (SER.DMP) and (1.8 + 0.04 and 9.7 + 0.05) minutes for both acidic degradation products of (SER.DMP), respectively, as shown in Figs. 2 and 3**.**

**Fig. 4:**
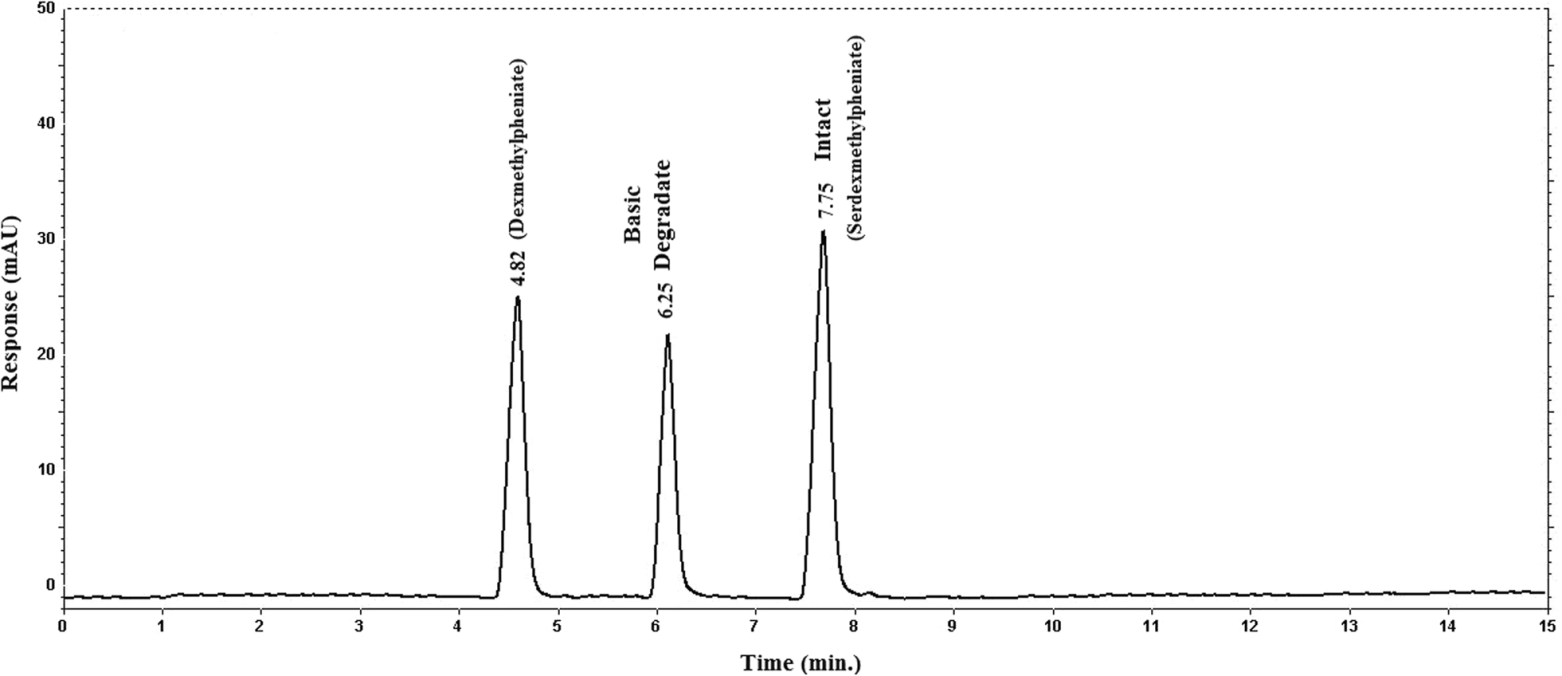
HPLC chromatogram of intact SER.DMP (10 µg/mL) and its basic degradates

On the other hand, the basic degradation of pure SER.DMP resulted in two basic degradants (Fig. 4: (A) 3-Carboxy-(R-1, 2-methoxy-2-oxo-1-phenylethyl) piperidine-1-Carbonyl-oxymethyl-pyridinium which converted to (DMP) and (B) L-Serine.

**Fig. 5:**
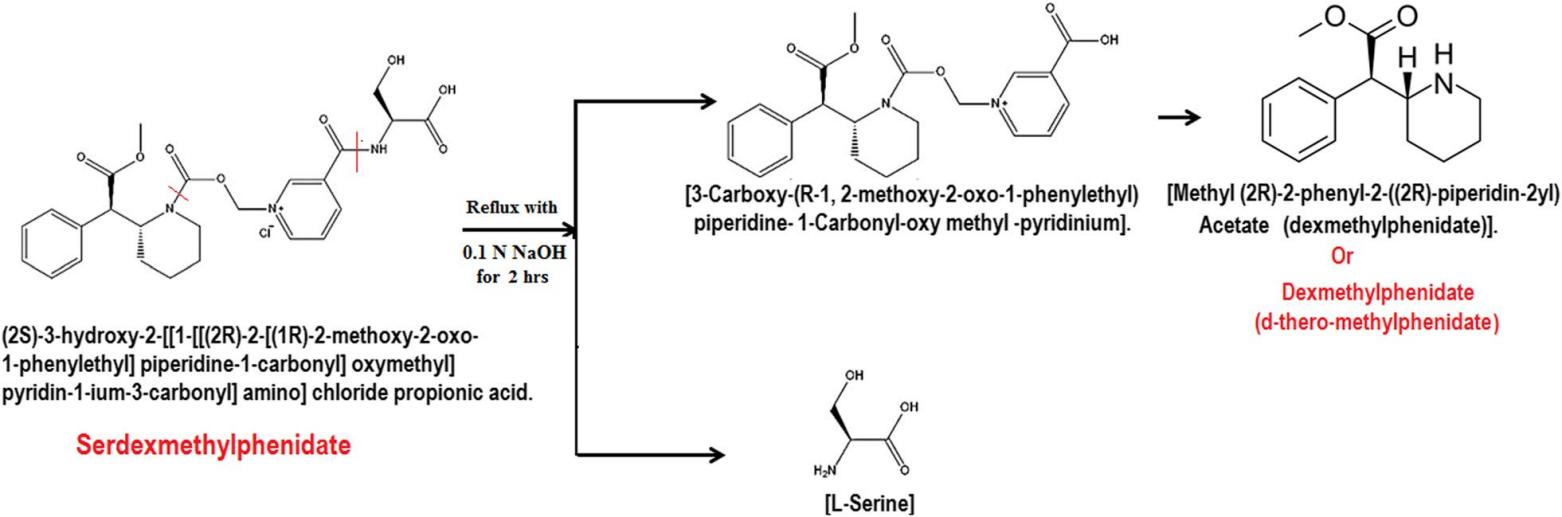
Suggested Basic degradation pathway of SER.DMP

**Fig. 6:**
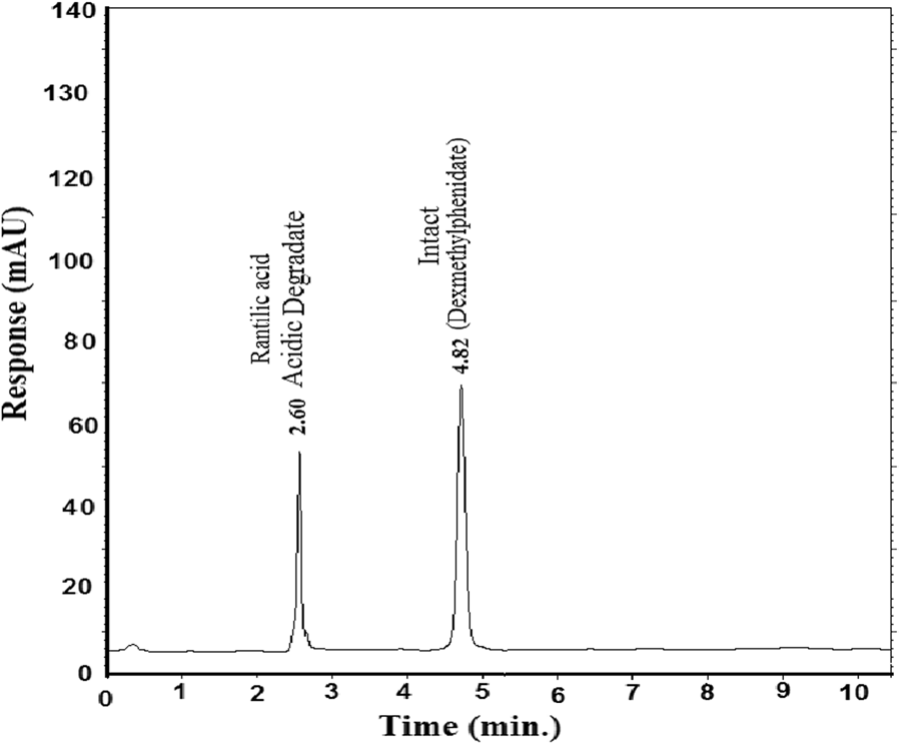
HPLC chromatogram of intact DMP (10 µg/mL) and its acidic degradant

**Fig. 7:**
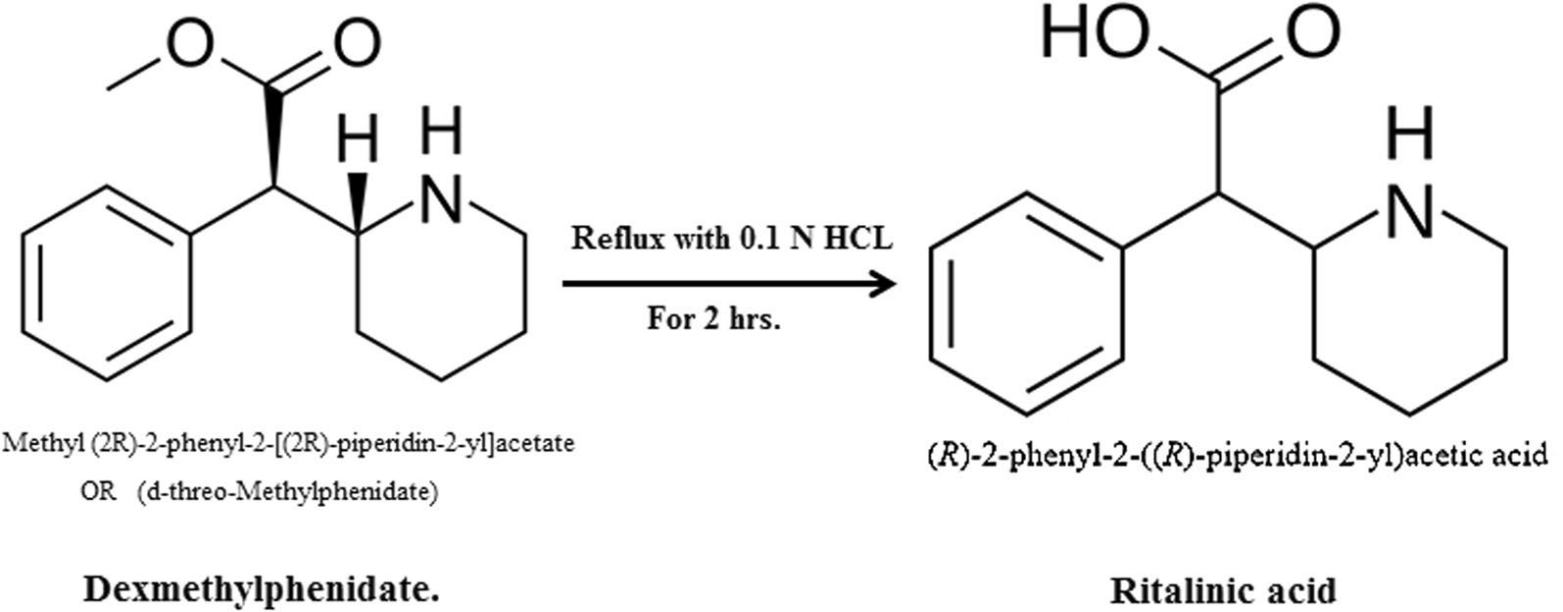
Suggested acidic degradation pathway of DMP

The HPLC chromatogram revealed that (DMP) and (SER.DMP) were clearly separated from the basic degradation products of (SER.DMP), at retention times (R_t_) of (4.8 + 0.02 and 7.7 + 0.05), respectively (Figs. 4, 5). The (DMP) acidic degradation resulted in one degradation product as per the HPLC chromatogram (Fig. 6). DMP was clearly separated from its acidic degradation product (Rantilic acid) at retention times of (4.8 + 0.02 and 2.6 + 0.05), respectively. Suggested acidic degradation pathway of DMP is presented in Fig. 7.

#### TLC densitometric method

**Fig. 8:**
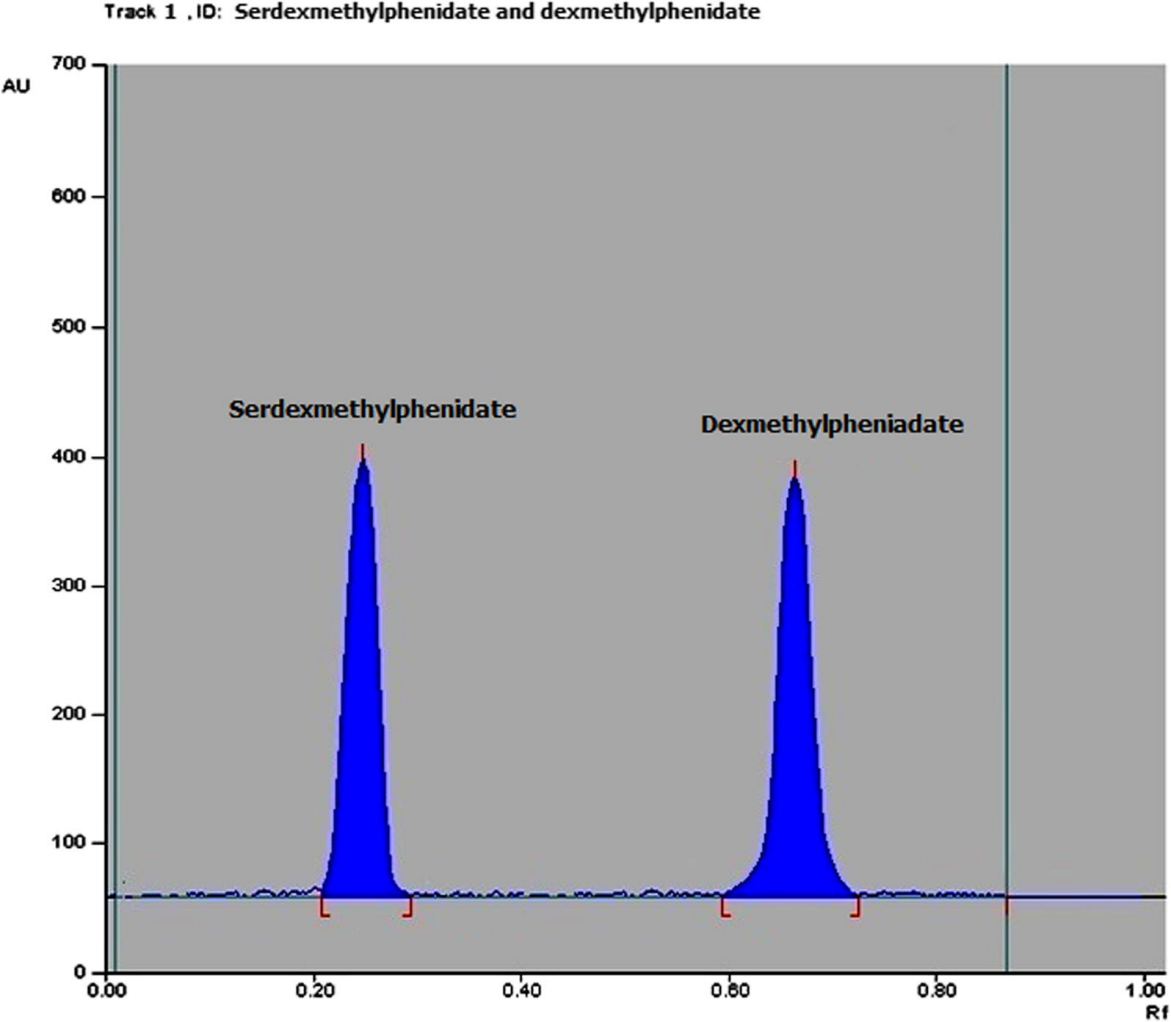
2D Densitometric chromatogram of SER.DMP and DMP mixture at 220 nm

**Fig. 9:**
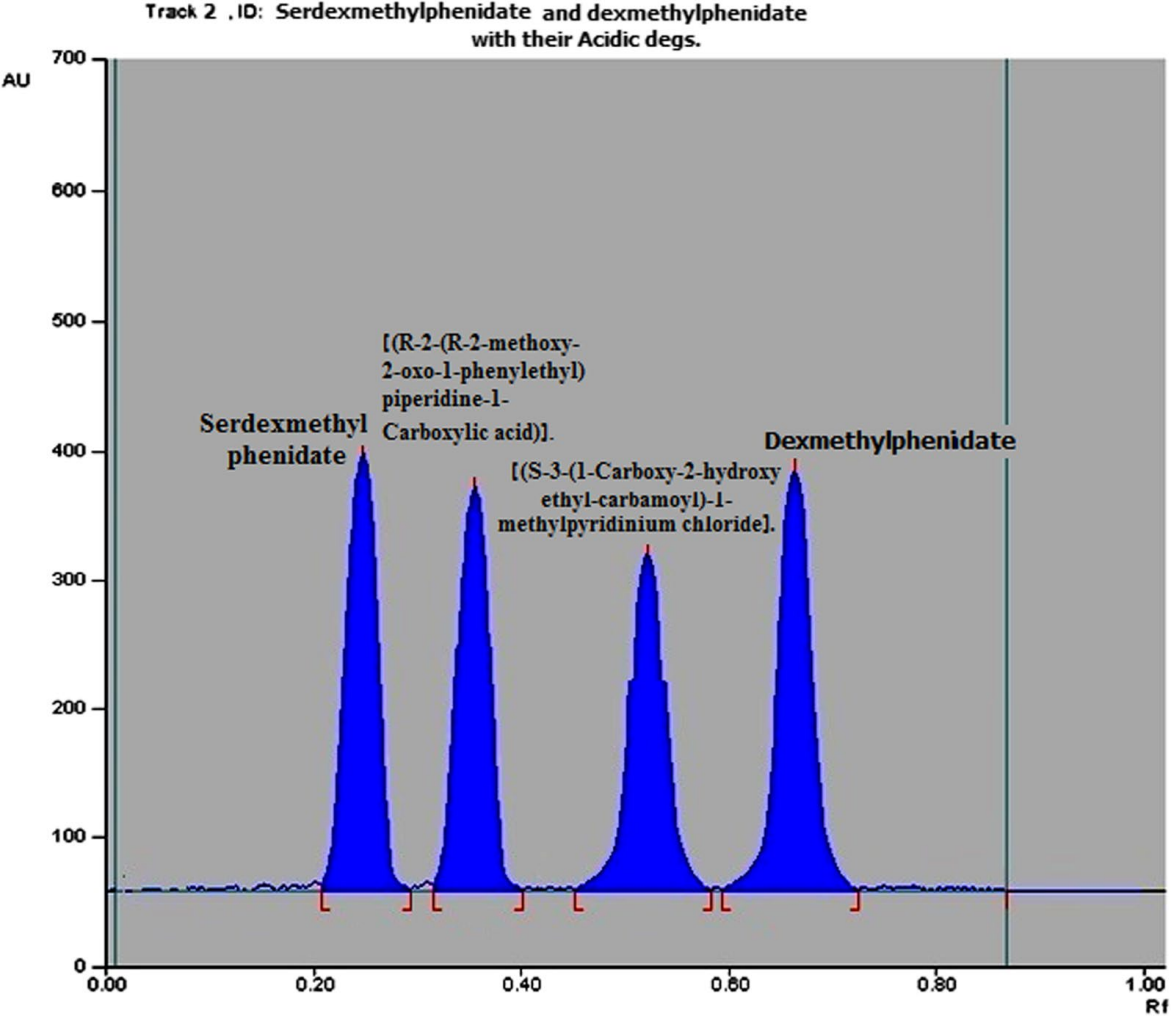
2D Densitometric chromatogram of SER.DMP and its acidic degradation products at 220 nm

**Fig. 10:**
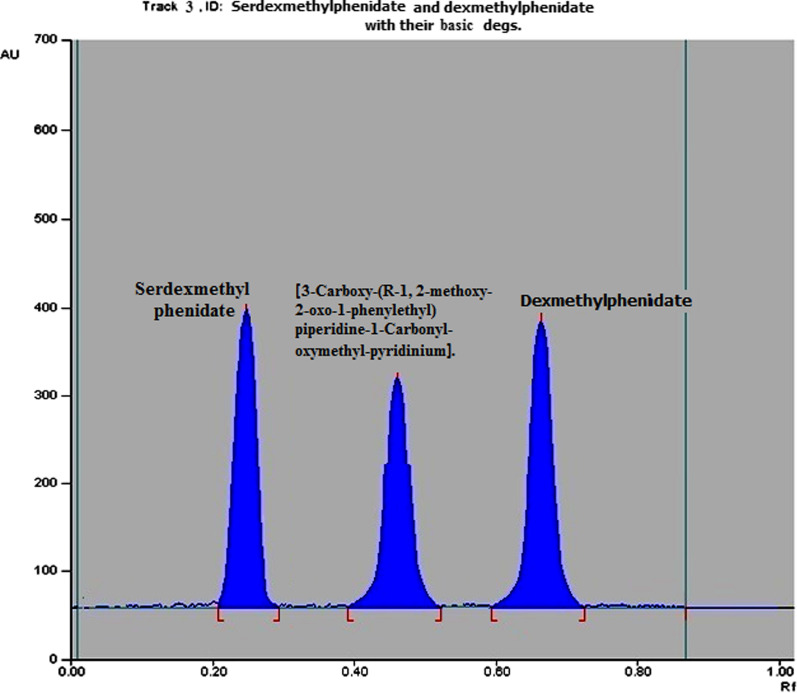
2D Densitometric chromatogram of SER.DMP and its basic degradation products at 220 nm

TLC densitometric method was applied to separate SER.DMP and DMP mixture using (methanol: chloroform 7:3 v/v) as a developing system and UV detection at 220 nm (Fig. 8 and Additional file 1: Fig. S3). The method was also applied to separate SER.DMP and DMP from their acidic and basic degradation products. The R_**f**_ values for SER.DMP and DMP were 0.24 and 0.62, respectively, whereas R_f_ values for SER.DMP acidic-induced degradation products were 0.35 and 0.57, respectively. The R_f_ value of its basic degradation product was 0.45 (Figs. 9, 10, Additional file 1: Fig. S4 and S5). This separation allows the determination of SER.DMP and DMP at 220 nm without any interference from its degradation product.

### Confirmation of complete acidic and basic degradation of SER.DMP

The degradation products formed under acidic and basic conditions were characterized through HPLC–DAD and TLC densitometry, isolation and subsequent infrared/nuclear magnetic resonance/mass spectral analyses and molecular dynamic simulation.

#### Confirmation of degradation products using TLC technique

Time required for complete degradation was determined by spotting on TLC plates every 30 min using mobile phase system (methanol: chloroform 70:30 v/v); complete degradation of SER.DMP was confirmed by absence of spot in the region corresponding to the spot of the intact drug.

#### Confirmation of degradation products using IR spectroscopy

IR spectrum of the entire SER.DMP (Additional file 1: Fig. S6), demonstrated peak of carbonyl group stretching in ester at 1730 which was shifted to 1710 cm^−1^ and peak of (-RC = ONH-) of amide group at (1470–1570) cm^−1^, while IR spectrum of acidic degradation product (Additional file 1: Figs. S7 and S8) demonstrated disappearance of (O-C = O) stretch of ester group and amide group (-RC = ONH-) and the presence of carboxylic group (-COOH) with very strong and broad peak at 3450 cm^−1^ (appears between 2800 and 3500 cm^−1^) for O–H stretch. The same IR spectrum of basic degradation product showed the same IR spectrum in addition to presence of amino group (-NH_2_) at (1630–1650) cm^−1^.

#### Confirmation of degradation product using ^1^H NMR spectroscopy

The ^1^H-NMR of the intact SER.DMP in dimethyl sulfoxide (DMSO) (Additional file 1: Fig. S9), showed triplet signal of three protons of aliphatic (-CH_3_) in ethyl group attached to nitrogen atom at 1.258–1.293 ppm, doublet signal of three protons of aliphatic (-CH_3_) attached to ethylene group at 1.975–1.998 ppm, singlet signal of three protons of aromatic (-CH_3_) attached to benzene ring at 2.239 ppm, multiplet signal of two protons of (-CH_2_-) in ethyl group attached to nitrogen atom at 4.227–4.280 ppm, multiplet signals of two protons of (-CH = CH-) in ethylene group at 6.251–6.607 ppm and multiplet signals of four aromatic protons at 7.045–7.567 ppm. The ^1^H-NMR of the acidic degradate in dimethyl sulfoxide (DMSO) (Additional file 1: Fig. S10), showed appearance of (-COOH-) carboxylic acid group singlets signal at 4.478 ppm which indicating cleavage of ester linkage with formation of carboxylic acid group. While the ^1^H-NMR of the basic degradate in dimethyl sulfoxide (DMSO) (Additional file 1: Fig. S11), showed appearance of (-NH-) secondary amino group singlet signal at 4.352 ppm indicating the cleavage of amide linkage with formation of amino group.

#### Confirmation of degradation products using mass spectrometry

Mass spectrometry showed intact SER.DMP with 535.98 molar mass and mixture of SER.DMP and DMP (Additional file 1: Figs. S12 and S13). SER.DMP acidic degradation revealed 3 degradants compounds: [Methyl (2R)-2-phenyl-2-((2R)-piperidin-2yl) Acetate (dexmethylphenidate)] molar mass was present at 233.32; [(R-2-(R-2-methoxy-2-oxo-1-phenylethyl) piperidine-1-Carboxylic acid)] molar mass was present at 277.32; and [(S-3-(1-Carboxy-2-hydroxyethyl-carbamoyl) -1-methyl pyridinium chloride], molar mass was present at 225.09 (Additional file 1: Fig. S14). The SER.DMP basic degradation resulted in 2 degradants compounds: [3-Carboxy-(R-1, 2-methoxy-2-oxo-1-phenylethyl) piperidine-1-Carbonyl-oxymethyl-pyridinium] molar mass was present at 413.45; and [L-Serine] molar mass was present at 105.09 (Additional file 1: Fig. S15).

### Molecular dynamic simulation

**Fig. 11:**
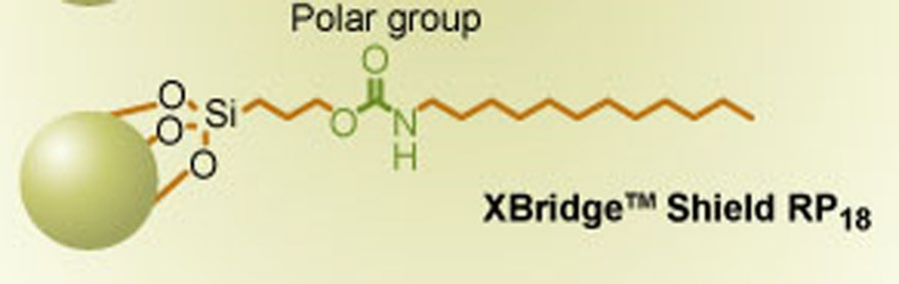
Diagram showing the composition of X-bridge shield RP_18_ column

The molecular dynamic simulation technique (MDST) is an important computational tool for understanding the system's dynamic development, determining the physical foundation of the structure, assessing the stability of interactions at the molecular level, and verifying practical work outcomes. As a result, the primary goal of using the molecular dynamic simulation methodology, which was employed for the first time in chromatographic approaches, was to evaluate the introduced drug's interaction with the stationary phase and to confirm the results of the proposed method by revealing which drug will be less retained in the stationary phase and which one will be more retained. Before conducting the simulation, DEC was adjusted at 51, which is equivalent to the total ratios of the mobile phase, as we cannot introduce the ratios of the solvent as it will give inaccurate simulation results. Here, the composition of the column plays a critical role in the interaction of the drugs with the stationary phase. The Reversed-phase C_18_ had only an octadecyl chain which could provide hydrophobic interactions; however, using X-bridge shield RP_18_, which offered the same interaction as reversed-phase C_18_, beside it will provide hydrogen bond donor, acceptor, and dipole interaction owing to the embedded carbamate (-O-CO-NH-) group (Fig. 11) in the bonded phase ligand that provided alternate selectivity, especially for phenolic compounds compared to straight-chain alkyl columns. Thus, in this work, we used an x-bridge column where the interactions between the drugs and the stationary phase were conducted, and the binding energies were calculated [8].

In the case of acidic degradation of SER.DMP, the resulting degradates and the intact drug were simulated under the mentioned conditions, and after a thorough examination of the resulting trajectories, we found that both degradates and the intact drug interact with the stationary phase in several different modes. For degradate1, a significant hydrogen bonding was noticed between its carbonyl groups and the carbamate moiety on the stationary phase with binding energies of − 3.5 and − 2.1 kcal/mol (Additional file 1: Figs. S16A and B). Also, a hydrophobic H-arene interaction between the aromatic ring in the degradate 1 and alkyl chain of the C18 stationary phase with binding energies of − 0.5 and − 0.6 kcal/mol (Additional file 1: Fig. S16C). DMP shows more powerful hydrogen bonding between its carbonyl, pyridinium nitrogen (NH2) and the stationary phase carbamate with binding energies of − 0.2 and − 8.2 kcal/mol (Additional file 1: Figs. S17A and B). Besides, a significant hydrophobic H-arene interaction is noticed between its aromatic ring and the alkyl chain of the C_18_ stationary phase with the binding energy of − 0.7 kcal/mol (Additional file 1: Fig. S17C). The intact drug molecule was found to form multiple stronger hydrogen bonds between the carboxyl and amino groups of its amide moiety and its terminal carboxylic (O) with the stationary phase carbamate groups with binding energies of − 1, − 1.8 and − 4.1 kcal/mol, respectively (Additional file 1: Figs. S18, A-C). Also, the intact drug was noticed to form hydrophobic H-arene interactions with the alkyl chain of the C18 stationary phase with binding energies of -0.8 kcal/mol (Additional file 1: Fig. S18D). While in the case of degradate 2, it forms multiple combined hydrogen bonds and hydrophobic arene interactions (Additional file 1: Figs. S19A–C). What is unique in the case of degradate 2 is the versatility of the binding modes, hydrogen bonding, besides the hydrophobic interactions, which made it the highest retained component.

In the case of basic degradation, the same methodology of simulations was conducted, and the trajectories were examined. The L-serine component showed only modest hydrogen bonding with stationary phase carbamate groups (Additional file 1: Fig. S20). While in the case of the basic degradation, moderate hydrogen bonding and hydrophobic interactions were noticed (Additional file 1: Fig. S21A–C).

Due to the diversity of the interactions between the analyzed components and the stationary phase, these observations were not enough to prove the arrangement of the separated components regarding their binding to the stationary phase. So, after conducting the molecular dynamics simulation, the solvation energies were calculated from the emerging trajectories. A calibration plot for each component in the acidic and basic degradations was generated by plotting the simulation time versus its solvation energy. Additional file 1: Figs. S22 and S23 arrange the acidic and basic degradations and the intact drug according to their solvation energies throughout the whole simulation time. The data extracted from the calculated solvation energies were in line with the binding energy data and experimental data from both the proposed chromatographic methods.

### Method validation

The development and validation of a stability-indicating assay method (SIAM) for any new drug substance and product requires complete information on the drug’s degradation behavior under a variety of stress conditions. It helps in establishing degradation pathways, allows separation and analysis of individual degradation products, and validates the stability indicating procedures used [12, 13]. A number of literature reports provide scientific rationale for the design of such studies [14–17] as little guidance is available on how to establish true ‘Selective’ stability-indicating methods.

A number of HPLC assay methods [5–7] were reported for determination of SER.DMP and DMP in pharmaceutical formulation and bulk powder. In these studies, partial degradation of the drug was accomplished using various forced degradation conditions. In one study [5], forced degradation was carried out by exposing dosage form to various stress conditions including HCl (0.1 N and 1 N) and NaOH (0.1 N and 1 N) at room temperature for 24 h to know the percentage degradation. Both drugs showed very little degradation (3.1–3.3%) in mild and (21.5–24.9%) in harsher acidic and basic stress. One degradation product was observed in HPLC chromatograms; the product was not isolated or identified, and its analytical profile was not established.

In the present investigation, complete degradation of both SER.DMP and DMP was achieved with 0.1N HCl, or 0.1N NaOH and reflux for 2 h at 60 °C and basic degradation of SER.DMP only by 0.1N NaOH at 60 °C for 2 h. Degradation products formed were characterized through HPLC–DAD and TLC densitometry and isolated by preparative TLC. Subsequently, infrared/nuclear magnetic resonance/mass spectral analyses and molecular dynamic simulation studies were performed to correlate the practical and the computational results and a validated Stability-Indicating Methods (HPLC and TLC densitometric) were developed.

The proposed methods were validated using the ICH guidelines [12, 13]. The validation results are shown in.

Table 1.

**Table 1:** Regression and validation data for the determination of SER.DMP and DMP by the proposed methods

Drug Name	SER.DMP	
Parameters	HPLC method	TLC densitometric method
Wavelength (nm)	220 nm	220 nm
Linearity range	2.5— 25 (μg/mL)	5— 25 (μg/spot)
LOD	0.051(μg/mL)	0.184 (μg/spot)
LOQ	0.165 (μg/mL)	0.202 (μg/spot)
Regression Equation	y ^a^ = 0.0989 C ^b^ + 0.0331	y ^a^ = 0.0824 C ^b^ + 0.0216
Correlation coefficient (r)	0.9998	0.9997
Accuracy (% R)** ± **SD	100.43 ± 1.02	99.68 ± 1.03
Precision (% RSD)		
Repeatability ^c^	1.021	1.025
Intermediate precision ^d^	1.015	1.039
Robustness (% RSD)		
Mobile phase contents ratio (± 2%)	0.812	1.386
Detection wavelengths (± 2 nm)	1.349	1.526
Flow rate (± 0.1 ml/min.)	1.778	**ـــــــ**

Drug Name	DMP	
Parameters	HPLC method	TLC densitometric method
Wavelength (nm)	220 nm	220 nm
Linearity range	2.5–25 (μg/mL)	5–25 (μg/spot)
LOD	0.098 (μg/mL)	0.115 (μg/spot)
LOQ	0.186 (μg/mL)	0.237 (μg/spot)
Regression Equation	y ^a^ = 0.0897 C ^b^ + 0.0522	y ^a^ = 0.0905 C ^b^ + 0.0612
Correlation coefficient (r)	0.9997	0.9996
Accuracy (% R) ± SD	100.15 ± 1.1	99.50 ± 1.2
Precision (% RSD)		
Repeatability ^c^	1.105	1.160
Intermediate precision ^d^	1.251	1.249
Robustness (% RSD)		
Mobile phase contents ratio (± 2%)	0.812	1.128
Detection wavelengths (± 2 nm)	1.349	1.630
Flow rate (± 0.1 ml/min.)	1.778	**ـــــــ**

^a^ Peak area of mixture of SER.DMP and DMP

^b^ Concentration in μg/ml for HPLC method and in μg/spot for TLC densitometric method

^c^ The intraday (n = 3), average of three concentrations of SER.DMP and DMP (10, 15 and

20 μg/mL) for HPLC method and (10,15 and 20 μg/spot) for TLC densitometric method

repeated three times within the day

^d^ The interday (n = 3), average of three concentrations of SER.DMP and DMP (10,15

and 20 μg/mL) for HPLC method and (10,15 and 20 μg/spot) for TLC densitometric

method repeated three times in three days

#### Linearity and range

Calibration graphs were constructed by plotting the peak area versus drug concentrations. The regression plots were found to be linear over the range of 2.5–25 µg/mL for RP-HPLC method and 5–25 µg/ spot for TLC-densitometric method.

The linear regression equations for serdexmethylphenidate graphs were:

$${\text{y}}{\mkern 1mu} \, = \,{\mkern 1mu} 0.0989{\mkern 1mu} {\text{x}}\,{\mkern 1mu} + \,{\mkern 1mu} 0.0331\quad \left( {r{\mkern 1mu} = {\mkern 1mu} 0.9998} \right),{\mkern 1mu} \,{\text{for}}\,{\mkern 1mu} {\text{RP}} - {\text{HPLC}}{\mkern 1mu} {\text{method}}.$$

$$\quad {\text{y}} \, = \, {\text{0}}.{\text{0824}} \, {\text{x}} \, + \, {\text{0}}.{\text{0216}}\quad \left( {{\text{r}} \, = \, {\text{0}}.{\text{9997}}} \right), \, {\text{for}} \, {\text{TLC}} - {\text{densitometric}} \, {\text{method}}.$$

The linear regression equations for dexmethylphenidate graph was:

$$\quad {\text{y}}^{{}} = \, {\text{0}}.{\text{0897}} \, {\text{x}} + \, {\text{0}}.{\text{0522}}\quad \left( {{\text{r}} \, = \, {\text{0}}.{\text{9996}}} \right), \, {\text{for}} \, {\text{RP}} - {\text{HPLC}} \, {\text{method}}.$$

$$\quad {\text{y}} \, = \, {\text{0}}.{\text{0905}} \, {\text{x}} + \, {\text{0}}.{\text{0612}}\quad \left( {{\text{r}} \, = \, {\text{0}}.{\text{9995}}} \right), \, {\text{for}} \, {\text{TLC}} - {\text{densitometric}} \, {\text{method}}.$$

where ***y*** is the area under peak values, ***x*** is the drug concentration and ***r*** is the correlation coefficient [18]. Linearity range, regression equation, intercept, slope and correlation coefficient for the calibration data were presented in Table 1.

#### LOD and LOQ

The limits of detection (LOD) and the limits of quantitation (LOQ) were calculated according to ICH guidelines from the following equations:

$${\text{LOD }} = { 3}.{3 }\sigma /{\text{S}}\quad {\text{LOQ }} = { 1}0 \, \sigma /{\text{S}}$$

where σ is the residual standard deviation of a regression line and S is the slope of the calibration curve [12]. LOD and LOQ values are given in Table 1.

#### Sensitivity

The proposed methods could determine both drugs at the low concentrations of (2.5 and 25 μg/mL), Table 1.

#### Accuracy and precision

Accuracy and precision of the methods were determined by applying the proposed procedure for determination of three different concentrations, each in triplicate of SER.DMP and DMP in pure form within linearity range (10,15,20 µg/mL or spot) in the same day (intraday) and in three successive days (interday). Accuracy as percent recovery (% R) and precision as percent relative standard deviation (%RSD) were calculated and results are listed in Table 1.

#### Stability of standard solutions

The stability of standard solutions of SER.DMP and its acidic and basic degradates were determined by repeated analysis of solutions stored either at room temperature or in refrigerator at different time intervals and comparing the responses (peak areas) with those of freshly prepared standard solutions. From the results obtained, both SER.DMP and DMP were stable for at least 4 days at room temperature and 7 days in refrigerator.

#### Specificity

The proposed methods were capable of determining SER.DMP and DMP selectively in its pharmaceutical formulation even in the presence of their degradants. Laboratory prepared mixtures of the intact drugs mixture of variable concentration ranging from 2.5–25 µg/mL together with different ratios of the basic and acidic degradation products of SER.DMP and acidic degradation product of DMP were prepared, the results obtained showed good recoveries up to 80% of acidic and 70% of basic degradants (Tables 2, 3, 4).

**Table 2:** Determination of SER.DMP in laboratory prepared mixtures with its acidic degradation products by the proposed HPLC and TLC procedures

SER.DMP. (HPLC method)				
Intact (µg/mL)	Acidic degradates (µg/mL)	Degradate %	Intact found (µg/mL)	Recovery % of Intact^a^
5	20	80	5.04	100.86
10	15	60	10.13	101.25
12.5	12.5	50	12.61	100.91
15	10	40	14.92	99.45
20	5	20	19.90	99.52
Mean				100.40
RSD%				0.843

SER.DMP. (TLC densitometric method)				
Intact (µg/spot)	Acidic degradates (µg/spot)	Degradate %	Intact found (µg/spot)	Recovery % of Intact^a^
5	20	80	4.99	99.96
10	15	60	10.07	100.07
12.5	12.5	50	12.51	100.21
15	10	40	14.98	99.95
20	5	20	19.60	99.85
Mean				100.01
RSD%				0.967

^a^Each experiment is repeated three times

**Table 3:** Determination of DMP in laboratory prepared mixtures with its acidic degradation products by the developed HPLC and TLC procedures

DMP (HPLC method)				
Intact (µg/ml)	Acidic degradates (µg/mL)	Degradate %	Intact found (µg/mL)	Recovery % of Intact^a^
5	20	80	5.01	100.20
10	15	60	10.05	100.5
12.5	12.5	50	12.51	100.08
15	10	40	14.97	99.80
20	5	20	19.95	99.75
Mean				100.06
RSD%				0.918

DMP (TLC densitometric method)				
Intact (µg/spot)	Acidic degradates (µg/spot)	Degradate %	Intact found (µg/spot)	Recovery % of Intact^a^
5	20	80	4.95	99.00
10	15	60	10.01	100.10
12.5	12.5	50	12.53	100.24
15	10	40	14.99	99.93
20	5	20	19.90	99.5
Mean				99.75
RSD%				0.989

^a^Each experiment is repeated three times

**Table 4:** Determination of SER.DMP in laboratory prepared mixtures with its basic degradation products by the developed HPLC and TLC procedures

HPLC method				
Intact (µg/mL)	Basic degradates (µg/mL)	Degradate %	Intact found (µg/mL)	Recovery % of Intact^a^
7.5	17.5	70	7.49	99.86
10	15	60	10.05	100.50
12.5	12.5	50	12.45	99.60
17.5	7.5	30	17.48	99.88
20	5	20	19.98	99.90
Mean				99.94
RSD%				0.911

TLC densitometric method				
Intact (µg/spot)	Basic degradates (µg/spot)	Degradate %	Intact found (µg/spot)	Recovery % of Intact^a^
7.5	17.5	70	7.48	99.73
10	15	60	9.95	99.50
12.5	12.5	50	12.52	100.16
17.5	7.5	30	17.49	99.94
20	5	20	19.95	99.75
Mean				99.82
RSD%				0.985

^a^Each experiment is repeated three times

The specificity of the stability indicating HPLC DAD method was assessed by resolving SER.DMP from its possible degradation products. The results revealed that the proposed methods were able to completely discriminate SER.DMP from all of its basic and acidic degradation products (acidic degradation was up to 80% of the SER.DMP drug and basic degradation was up to 70% of the SER.DMP), confirming the specificity of the method. Moreover, the peak purity was checked using DAD and the purity of SER.DMP was found to be more than 0.992 indicating that no additional peaks were co-eluted with the main compound.

The specificity of the TLC- densitometric method was assured by applying it to laboratory prepared mixtures of the intact SER.DMP together with its basic and acidic degradation products. The proposed procedure was adopted for the selective determination of intact SER.DMP and DMP in presence of its degradation product, as shown in Tables 2, 3, 4.

#### Sensitivity

The two proposed methods were capable of determining SER.DMP and DMP at low concentrations as showed in Table 1.

#### Robustness

The robustness of the methods was evaluated by minor changes in mobile phase ratios, flow rate and wavelength and did not have any significant effect on the %RSD of SER.DMP and DMP quantification; confirming the solidarities of both developing methods, as shown in Table 1.

#### Validity

The validity of the obtained results was assessed by the recovery of the added standards by standard addition technique (Table 5). Standard addition is done by spiking known amount of pure drug (powder) to a certain known amount of capsule (powder) then mixing and analyzing. The results are presented in Table 5.

**Table 5:** Application of standard addition technique for determination of SER.DMP and DMP in capsules using the developed chromatographic techniques

Pharmaceutical taken (µg/mL)	HPLC method			TLC densitometric method		
	Standard added ^b^(μg/mL)	% Recovery of added standard^a^		Standard added^b^ (μg/spot)	% Recovery of added standard^a^	
		SER.DMP	DMP		SER.DMP	DMP
15	10	101.25	99.75	10	100.07	99.95
10	15	99.45	100.69	15	99.95	100.75
5	20	99.52	99.82	20	99.85	99.92
Mean		100.27	100.43		99.95	100.31
% RSD		0.830	0.824		0.963	1.129

^a^Average of three different experiments

^b^Each of both drugs

### Statistical comparison with the reported methods

The obtained results were statistically compared to those obtained by the reported method [5] indicating good accuracy and precision of the proposed methods for the analysis of the studied drug in its pharmaceutical dosage form, as shown in Table 6. No significant differences were found by applying student’s *t*-test and *F*-test at 95% confidence level.

**Table 6:** Determination of SER.DMP and DMP in Azstarys^®^ capsules by the proposed methods and the reported method

Parameters	SER.DMP		
	HPLC method	TLC densitometric method	Reported method^a^ [5]
Number of measurements	5	5	5
Mean % recovery of SER.DMP	100.43	99.68	99.43
% RSD	0.910	1.085	1.380
Student’s t-test^b^	1.153 (2.306)	0.457 (2.306)	——
F-value^b^	2.260 (6.388)	1.606 (6.388)	——

Parameters	DMP		
	HPLC method	TLC densitometric method	Reported method^a^ [5]
Number of measurements	5	5	5
Mean % recovery of DMP	100.43	99.68	99.43
% RSD	0.910	1.085	1.380
Student’s t-test^b^	1.153 (2.306)	0.457 (2.306)	——
F-value^b^	2.260 (6.388)	1.606 (6.388)	——

^a^ Reference method is HPLC using X-terra C18 column using a mixture of trifluoro acetic acid and acetonitrile (70:30 v/v) as mobile phase with a flow rate of 1 mL and UV-detection at 265 nm

^b^ The values in parenthesis are tabulated values of “*t”* and “*F”* at (P = 0.05

### Application of developed methods to pharmaceutical preparation

The proposed methods were applied to the determination of SER.DMP and DMP in Azstarys^®^ capsules. Satisfactory results were obtained in good agreement with the label claim, indicating no interference from excipients and additives **(**Table 6).

### Comparison between published (Ref. 5) and newly developed methods

A comparison between published (Ref. 5) and newly developed methods with respect to Chromatographic Conditions, acid and alkaline degradation, method validation and verification of practical outcomes by Molecular Dynamic Simulation Technique (MDST) is provided in Table 7. The published method (Ref.5) is considered as a study of forced degradation under different stress conditions whereas the developed methods are validated stability-indicating assay chromatographic methods for the determination of both SER.DMP and DMP drug mixtures in presence of their acidic or alkaline degradation products.

**Table 7:** Comparison between published (Ref.5) and newly developed methods

Parameters	Published method (Ref.5)		Developed methods			
			HPLC		TLC- densitometric	
	SER.DMP	DMP	SER.DMP	DMP	SER.DMP	DMP
Chromatographic conditions						
Mobile phase	Trifluoroacetic acid: acetonitrile (70:30 v/v)		5 mM phosphate buffer (pH 5.5): acetonitrile (40:60 v/v)		methanol: chloroform (70:30 v/v)	
Detection wavelength	265 nm	265 nm	220 nm	220 nm	220 nm	220 nm
Run time	7.33 min	7.33 min	7.75 min	7.75 min	–-	–-
Retention time R_t_ or	2.71 min	7.33 min	7.75 min	4.82 min	–-	–-
Flow rate (R_f_)	–	–-	–	–-	0.24 min	0.62 min
Forced degradation (% degradation)						
HCl (0.1N)			–	–	–	–
Normal condition	3.1%	3.3%				
HCl (0.1N)						
Reflux for 2 h at 60 °C	–	–	100% verified by TLC, three degradants	100% verified by TLC, one degradant	100% verified by TLC, three degradants	100% verified by TLC, one degradant
HCl (1N)						
Normal condition	22%	21.5%	N/A			
NaOH (0.1N)						
Normal condition	2.7% %	2.5%	–	–	–	–
NaOH (0.1N)						
Reflux for 2 h at 60 °C	–	–	100% verified by TLC, two degradants	100% verified by TLC, two degradants	100% verified by TLC, two degradants	100% verified by TLC, two degradants
NaOH (1N)						
Normal condition	24.9%	20.3%	N/A			
Degradation products						
Separation						
Identification of acid degradants	–	–	+	+	+	+
Separation						
Identification of alkaline degradants	–	–	+	**–**	+	**–**
Confirmation of degradation pathway	–	–	confirmed using TLC, IR, ^1^H-NMR and mass spectroscopy			
Method validation						
Linearity range/sensitivity (μg/mL)	4.2–63	0.9to3.5	2.5–25	5–25	5–25	5–25
LOD (μg/mL)	0.042	0.009	0.051	0.098	0.184	0.115
LOQ (μg/mL)	0.42	0.09	0.165	0.186	0.202	0.237
Selectivity (application to lab prepared mixtures in presence of degradants)	Not tested	Not tested	No interference from degradation products,up to 80% acidic & 70% alkaline	No interference from degradation products, up to 80% ( acidic)	No interference from degradation products, up to 80% acidic & 70% alkaline	No interference from degradation products, up to 80% acidic
Application of standard addition technique^a^	–	–	+	+	–	–
Verification of practical outcomes by MDST^b^	–	–	+	+	–	–

^a^ Accuracy assessment

^b^ Molecular Dynamic Simulation Technique

## Conclusion

HPLC and TLC chromatographic methods were developed and applied for the simultaneous determination of SER.DMP and DMP in the presence of their degradation products, where good results with low LOD and LOQ were obtained. In addition, the practical HPLC work was supported by applying Computerized Computational full data. Both proposed stability indicating HPLC and TLC methods are simple, accurate and precise (ICH validation), so they can be applied for the determination of these drugs in pharmaceutical dosage forms in quality control laboratory.

## Supplementary Information

**Additional file 1:** **Fig. S1.** HPLC chromatograms of Serdexmethylphenidate (A) 2D at 220 nm (B) 3D chromatogram in scanning mode using DAD. **Fig. S2**. UV Absorption spectrum of 5 μg/mL of Serdexmethylphenidate. **Fig. S3.** 3D Densitometric chromatogram of Serdexmethylphenidate (2.5- 25µg/spot) at 220 nm. **Fig. S4.** 3D Densitometric chromatogram of Serdexmethylphenidate and Acidic induced degradation products at 220 nm. **Fig. S5**. 3D densitometric chromatogram of Serdexmethylphenidate and basic induced degradation product at 220 nm. **Fig. S6**. IR Spectrum of Serdexmethylphenidate on KBr disc. **Fig. S7**. IR Spectrum of Serdexmethylphenidate HCL degradates on KBr disc. **Fig. S8.** IR Spectrum of Serdexmethylphenidate NaOH degradates on KBr disc. **Fig. S9**. ^1^H-NMR Spectrum of Serdexmethylphenidate in DMSO. **Fig. S10**. ^1^H-NMR Spectrum of Acidic Degradates of Serdexmethylphenidate. **Fig. S11.** ^1^H-NMR Spectrum of Basic Degradates of Serdexmethylphenidate. **Fig. S12**. Mass Spectrum of Serdexmethylphenidate. **Fig. S13**. Mass Spectrum of Mixture Serdexmethylphenidate and dexmethylphenidate. **Fig. S14.** Mass Spectrum of Acidic Degradates of Serdexmethylphenidate. **Fig. S15**. Mass Spectrum of Basic Degradates of Serdexmethylphenidate. **Fig. S16**. 2D Interaction plot showing binding interactions and energies (A, B) between the carbonyl groups of degradate 1 and the carbamate moiety on the stationary phase via hydrogen bonding, and (C) between the aromatic ring in the degradate 1 and alkyl chain of the C_18_ stationary phase via a hydrophobic H-arene interaction. **Fig. S17**. 2D Interaction plot showing binding interactions and energies (A,B) between carbonyl, pyridinium nitrogen of Dexmethylphenidate and the stationary phase carbamate via hydrogen bonding, and (C) between the aromatic ring of Dexmethylphenidate and alkyl chain of the C_18_ stationary phase via a hydrophobic H-arene interaction. **Fig. S18**. 2D Interaction plot showing binding interactions and energies (A-C) between the carboxyl and amino groups of the amide moiety and terminal carboxylic of Serdexmethylphenidate with the stationary phase carbamate groups via hydrogen bonding and (D) between the aromatic ring of Serdexmethylphenidate and alkyl chain of the C_18_ stationary phase via a hydrophobic H-arene interaction. **Fig. S19**. (A-C) 2D Interaction plot showing binding interactions and energies between degradate 2, and stationary phase via hydrogen bonding and hydrophobic arene interactions. **Fig. S20**. 2D Interaction plot showing binding interactions and energies between L-serine and the stationary phase carbamate groups via hydrogen bonding. **Fig. S21**. (A-C) 2D Interaction plot showing binding interactions and energies between basic degradate, and stationary phase via hydrogen bonding and hydrophobic arene interactions. **Fig. S22**. A calibration plot for each component in the acidic degradation showing the simulation time versus its solvation energy. **Fig. S23**. A calibration plot for each component in the basic degradation showing the simulation time versus its solvation energy.

## Data Availability

All data and materials generated or analyzed during this study are included in this published article and its supplementary information files.
