# Supplementary material for: Chromatographic reversed HPLC and TLC-densitometry methods for simultaneous determination of serdexmethylphenidate and dexmethylphenidate in presence of their degradation products—with computational assessment
Source: BMC Chem. 2023 Jul 15;17(1):76. doi: 10.1186/s13065-023-00986-3 (PMC10349413; doi:10.1186/s13065-023-00986-3)
Supplement: Supplementary file 1 — Additional file 1: Fig. S1. HPLC chromatograms of Serdexmethylphenidate (A) 2D at 220 nm (B) 3D chromatogram in scanning mode using DAD. Fig. S2. UV Absorption spectrum of 5 μg/mL of Serdexmethylphenidate. Fig. S3. 3D Densitometric chromatogram of Serdexmethylphenidate (2.5- 25µg/spot) at 220 nm. Fig. S4. 3D Densitometric chromatogram of Serdexmethylphenidate and Acidic induced degradation products at 220 nm. Fig. S5. 3D densitometric chromatogram of Serdexmethylphenidate and basic induced degradation product at 220 nm. Fig. S6. IR Spectrum of Serdexmethylphenidate on KBr disc. Fig. S7. IR Spectrum of Serdexmethylphenidate HCL degradates on KBr disc. Fig. S8. IR Spectrum of Serdexmethylphenidate NaOH degradates on KBr disc. Fig. S9. 1H-NMR Spectrum of Serdexmethylphenidate in DMSO. Fig. S10. 1H-NMR Spectrum of Acidic Degradates of Serdexmethylphenidate. Fig. S11. 1H-NMR Spectrum of Basic Degradates of Serdexmethylphenidate. Fig. S12. Mass Spectrum of Serdexmethylphenidate. Fig. S13. Mass Spectrum of Mixture Serdexmethylphenidate and dexmethylphenidate. Fig. S14. Mass Spectrum of Acidic Degradates of Serdexmethylphenidate. Fig. S15. Mass Spectrum of Basic Degradates of Serdexmethylphenidate. Fig. S16. 2D Interaction plot showing binding interactions and energies (A, B) between the carbonyl groups of degradate 1 and the carbamate moiety on the stationary phase via hydrogen bonding, and (C) between the aromatic ring in the degradate 1 and alkyl chain of the C18 stationary phase via a hydrophobic H-arene interaction. Fig. S17. 2D Interaction plot showing binding interactions and energies (A,B) between carbonyl, pyridinium nitrogen of Dexmethylphenidate and the stationary phase carbamate via hydrogen bonding, and (C) between the aromatic ring of Dexmethylphenidate and alkyl chain of the C18 stationary phase via a hydrophobic H-arene interaction. Fig. S18. 2D Interaction plot showing binding interactions and energies (A-C) between the carboxyl and amino groups of the amid [file 13065_2023_986_MOESM1_ESM.docx]

**Addition File**

**Chromatographic Reversed HPLC and TLC-Densitometry Methods for Simultaneous Determination of Serdexmethylphenidate and Dexmethylphenidate in Presence of their Degradation Products - with Computational Assessment**


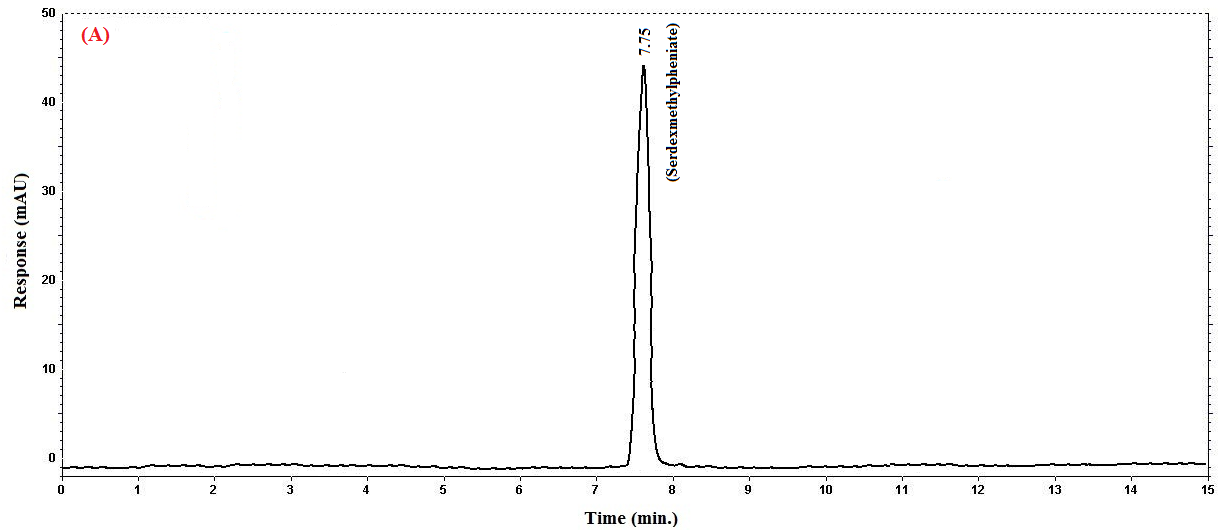


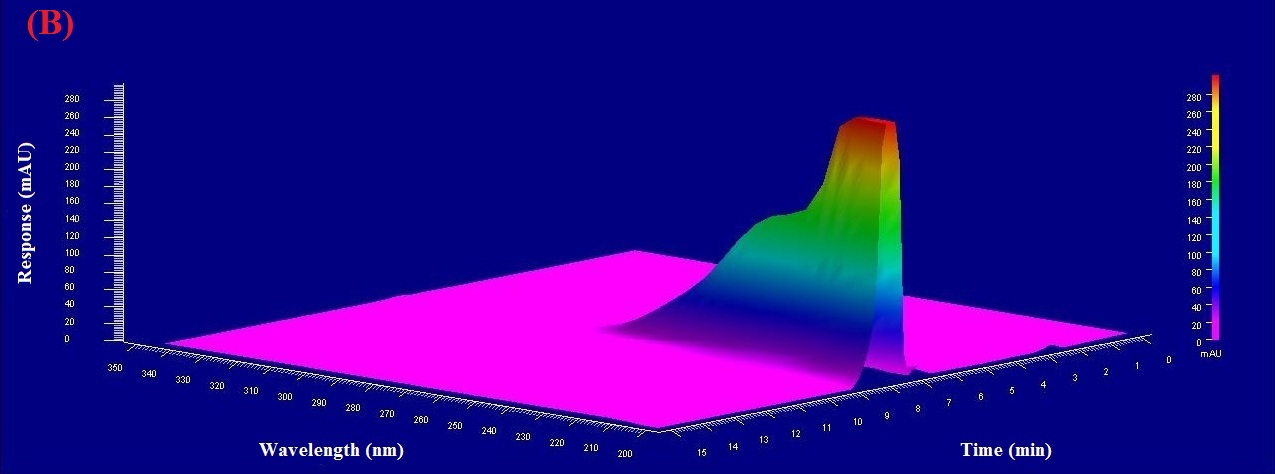


**Fig. S1. HPLC chromatograms of Serdexmethylphenidate**

**(A) 2D at 220 nm**

**(B) 3D chromatogram in scanning mode using DAD**


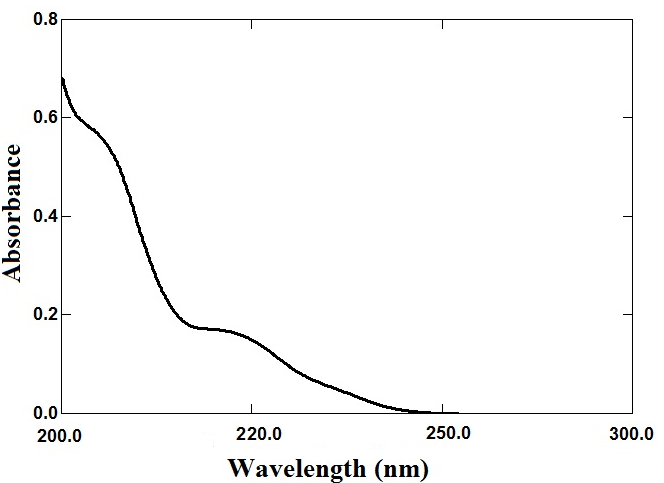


**Fig. S2. UV Absorption spectrum of 5 μg/mL of Serdexmethylphenidate**


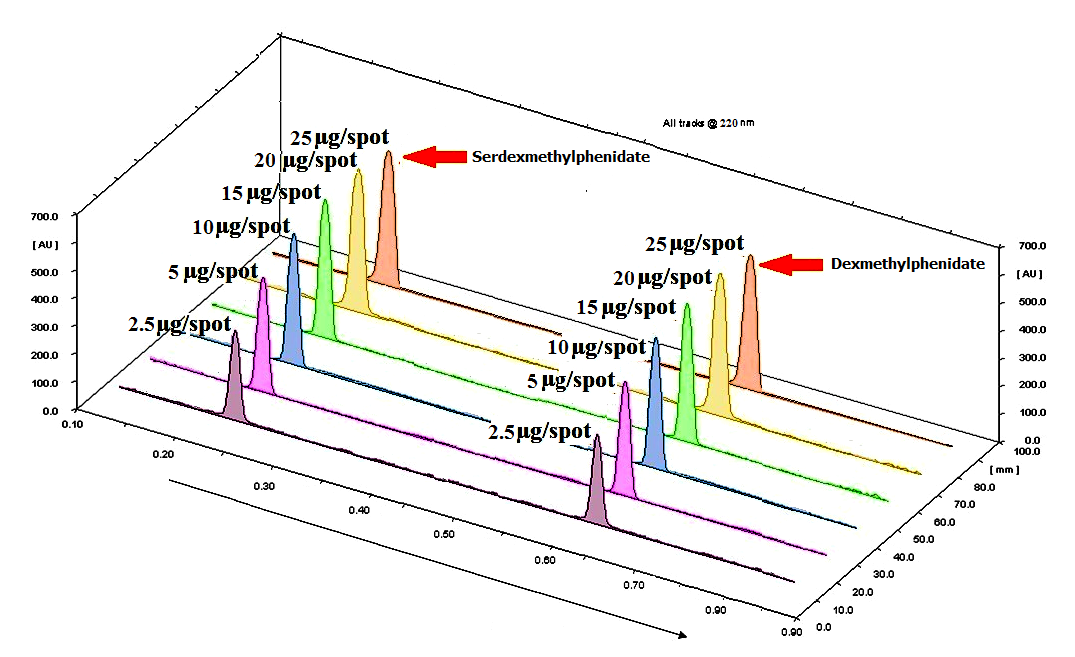


**Fig. S3. 3D Densitometric chromatogram of Serdexmethylphenidate**

**(2.5- 25µg/spot) at 220 nm**

**
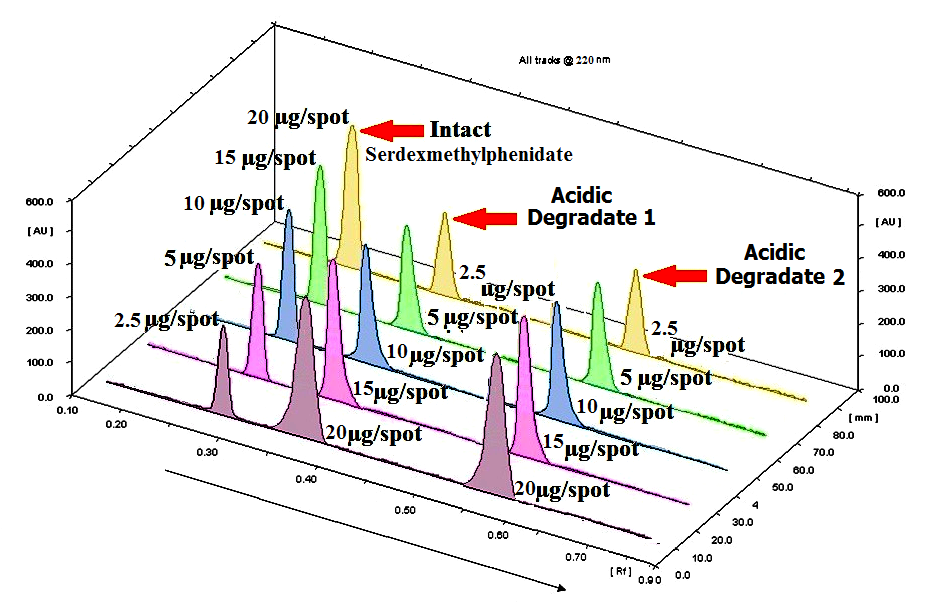
**

**Fig. S4. 3D Densitometric chromatogram of Serdexmethylphenidate**

**and Acidic induced degradation products at 220 nm**

**
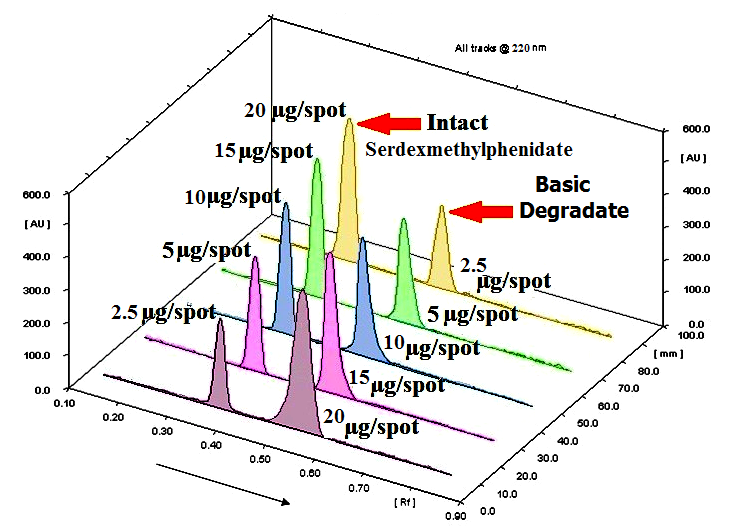
**

**Fig. S5. 3D densitometric chromatogram of Serdexmethylphenidate**

**and basic induced degradation product at 220 nm**


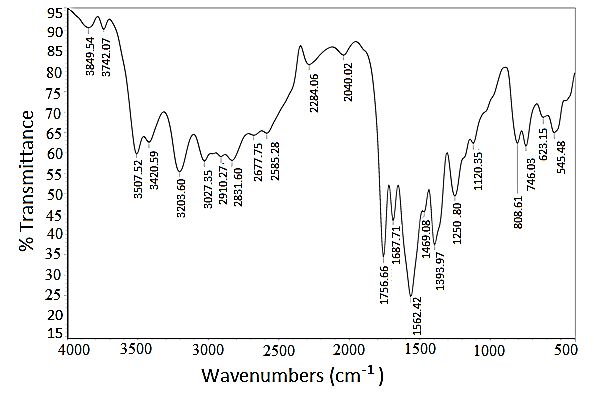


**Fig. S6. IR Spectrum of Serdexmethylphenidate on KBr disc**

*
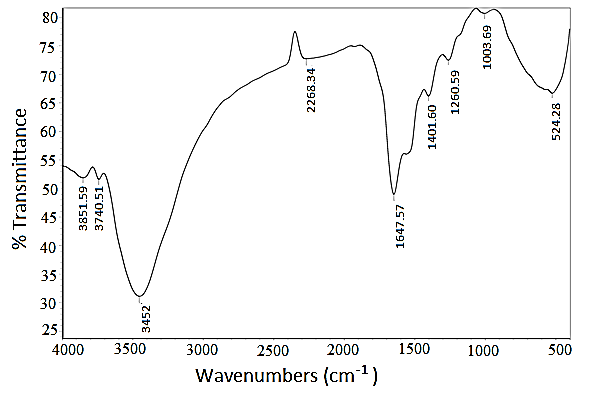
*

**Fig. S7. IR Spectrum of Serdexmethylphenidate HCL degradates on KBr disc**

***
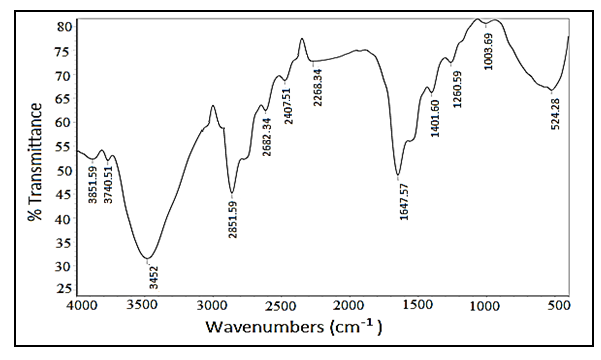
***

**Fig. S8. IR Spectrum of Serdexmethylphenidate NaOH degradates on KBr disc**


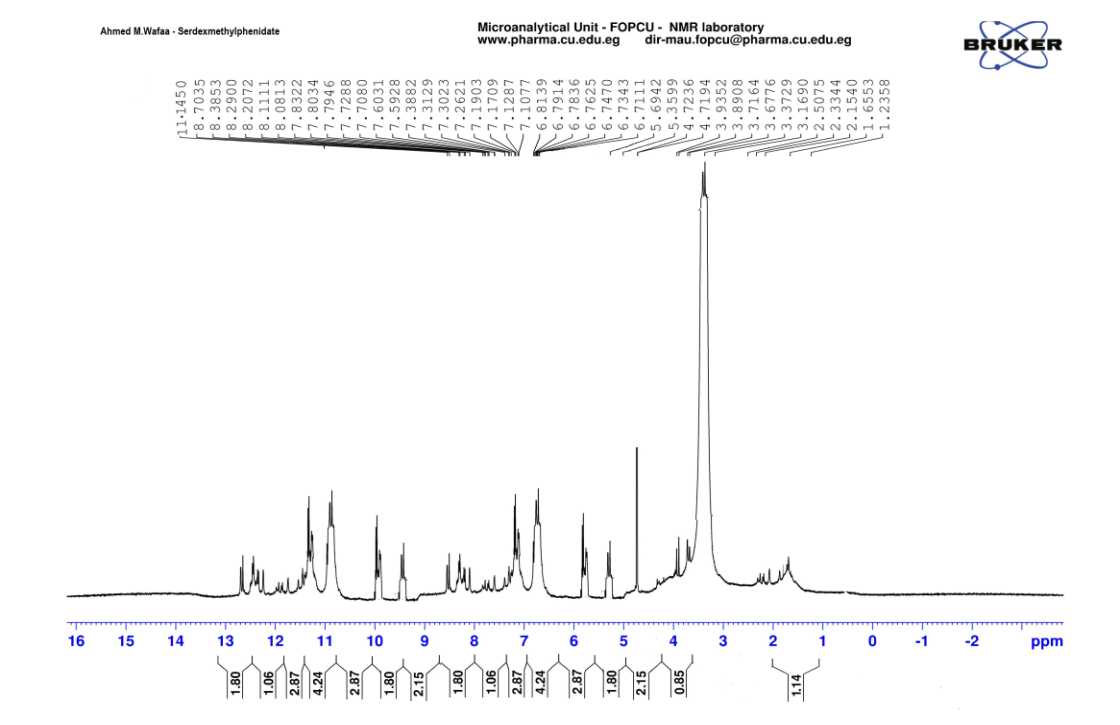


**Fig. S9. ^1^H-NMR Spectrum of Serdexmethylphenidate in DMSO**


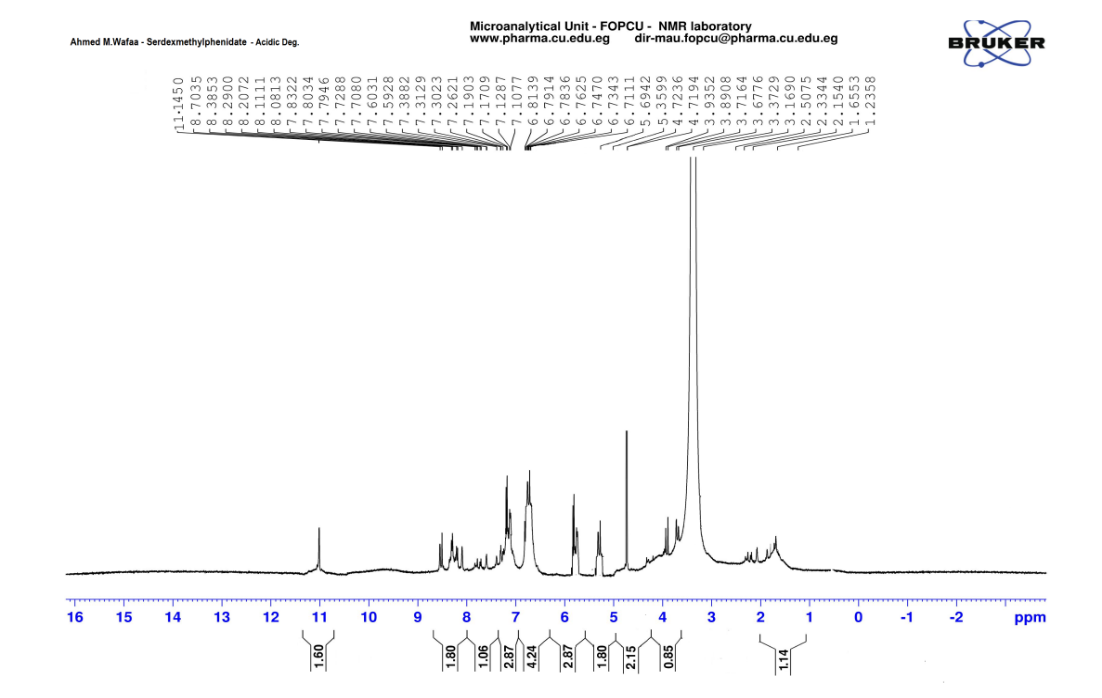


**Fig. S10. ^1^H-NMR Spectrum of Acidic Degradates of Serdexmethylphenidate**


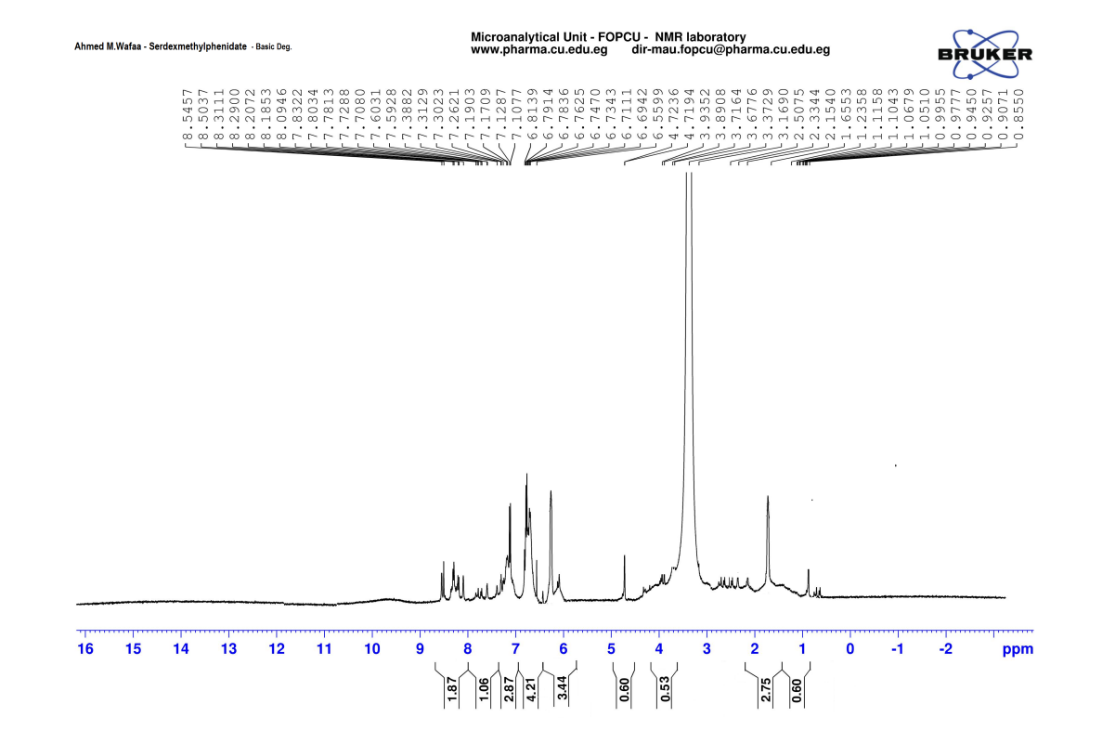


**Fig. S11. ^1^H-NMR Spectrum of Basic Degradates of Serdexmethylphenidate**


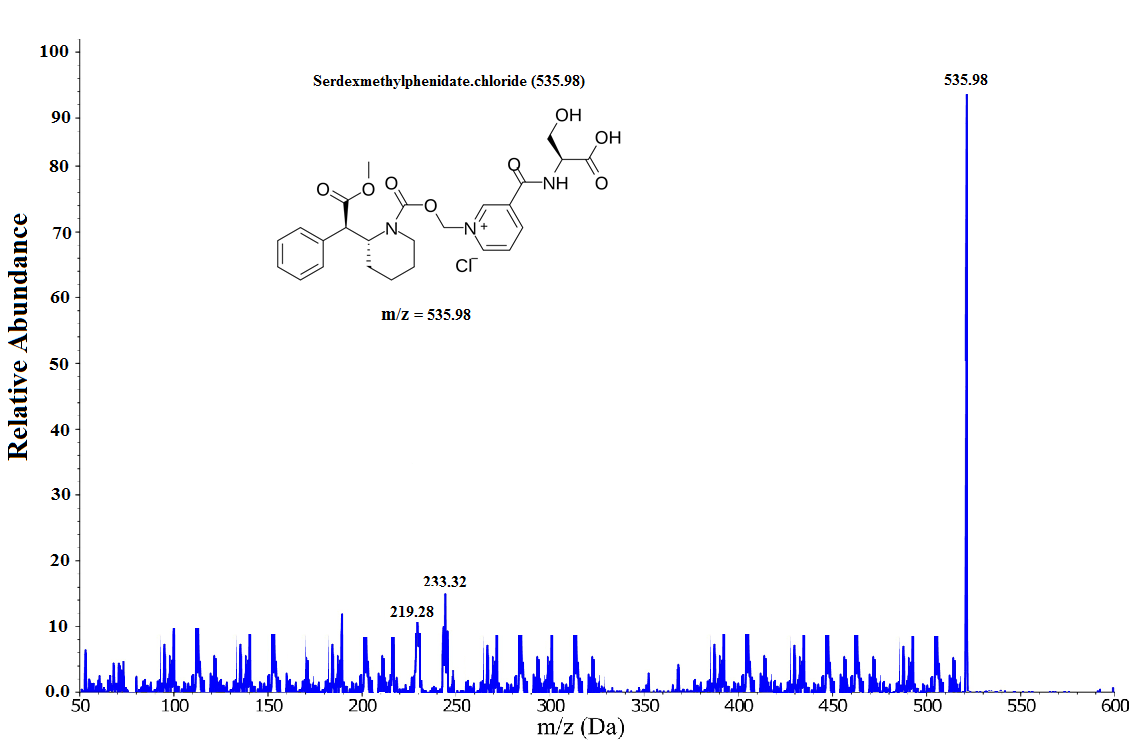


**Fig. S12. Mass Spectrum of Serdexmethylphenidate**


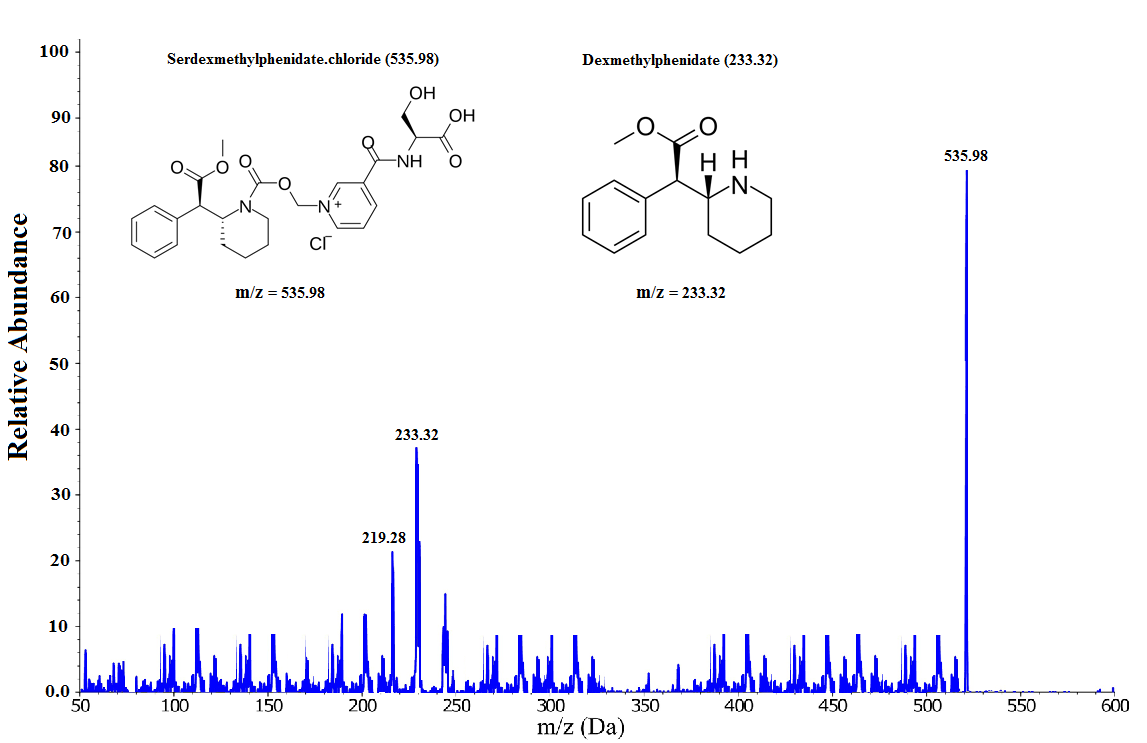


**Fig. S13. Mass Spectrum of Mixture Serdexmethylphenidate and dexmethylphenidate**


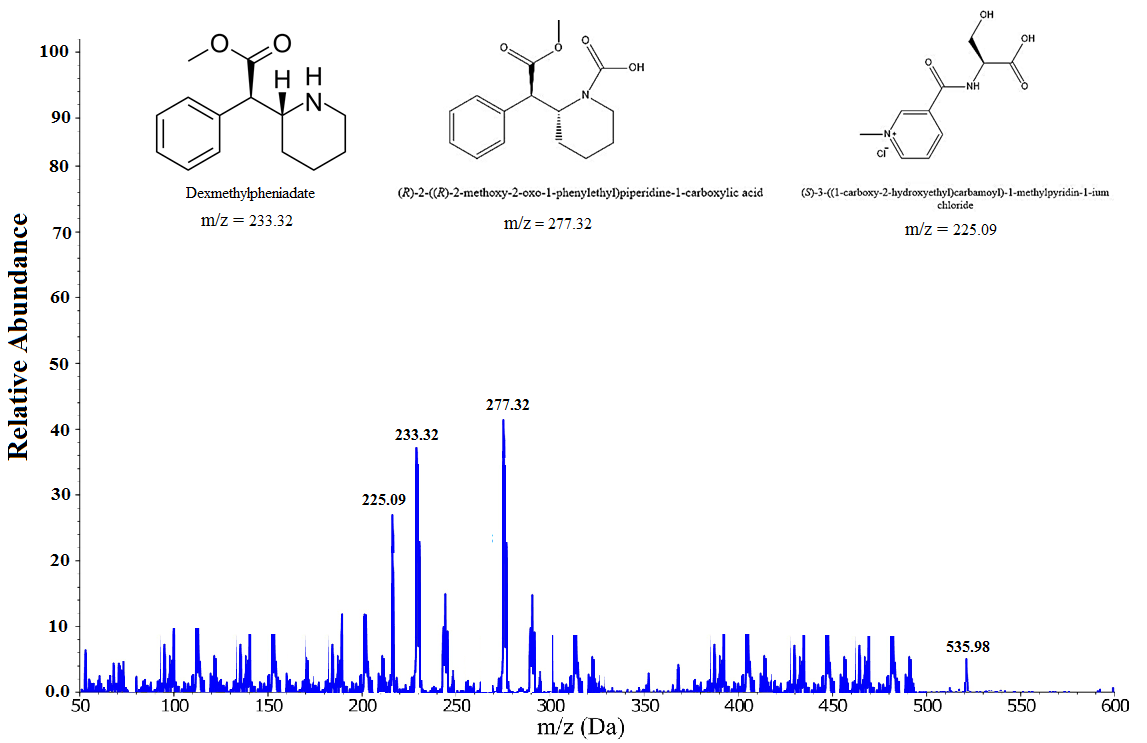


**Fig. S14. Mass Spectrum of Acidic Degradates of Serdexmethylphenidate**


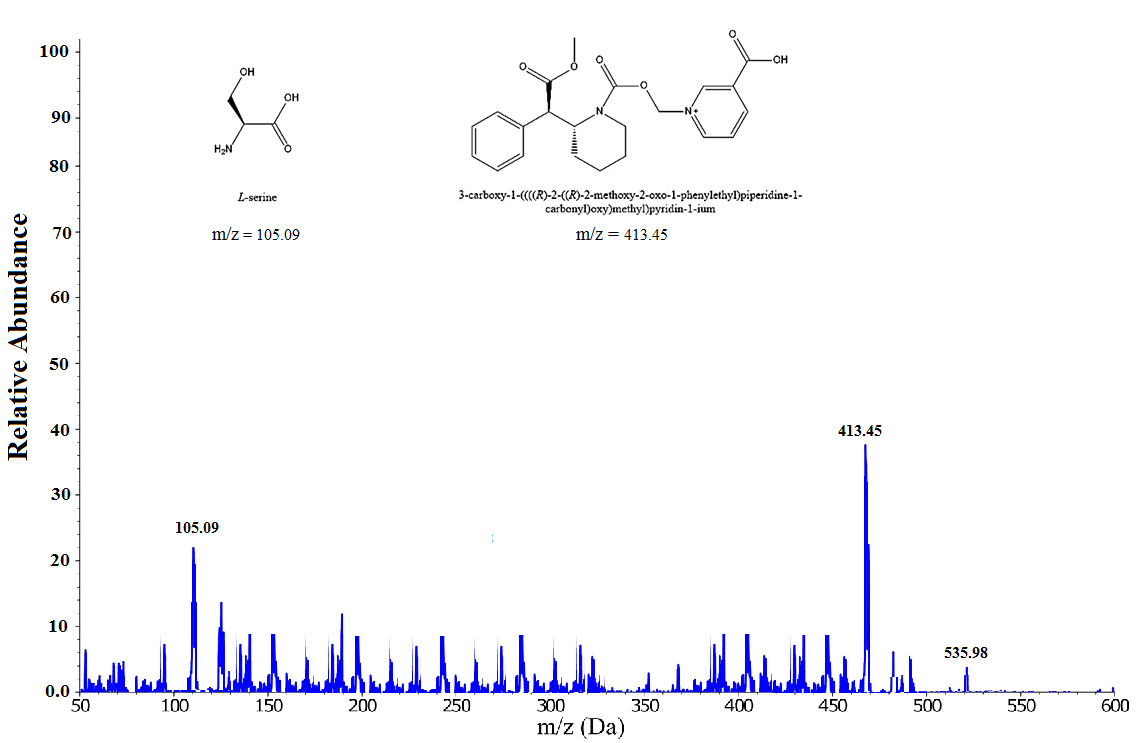


**Fig. S15. Mass Spectrum of Basic Degradates of Serdexmethylphenidate**

**
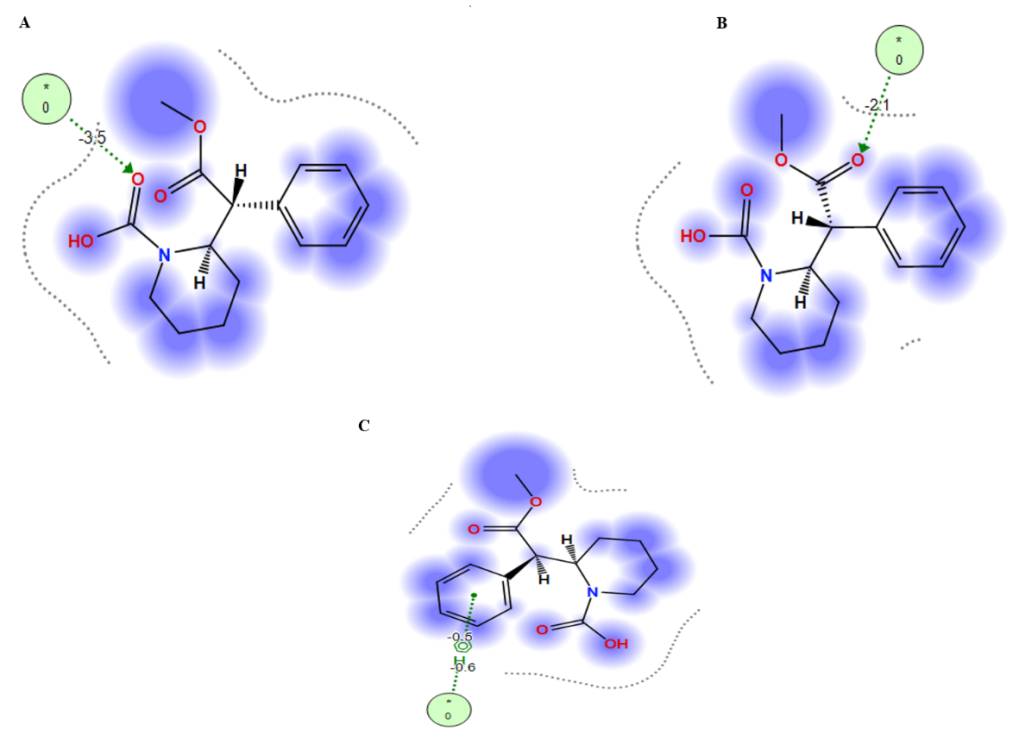
**

**Fig. S16. 2D Interaction plot showing binding interactions and energies**

**(A, B) between the carbonyl groups of degradate 1 and the carbamate moiety**

**on the stationary phase via hydrogen bonding, and (C) between**

**the aromatic ring in the degradate 1 and alkyl chain of the**

**C_18_ stationary phase via a hydrophobic H-arene interaction**

**
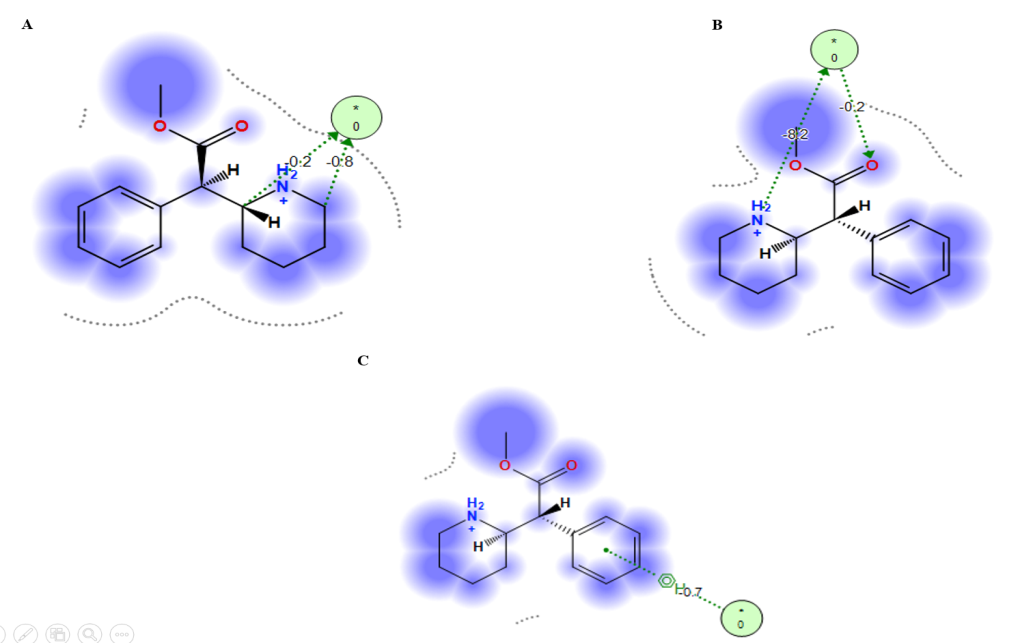
**

**Fig. S17. 2D Interaction plot showing binding interactions and energies**

**(A,B) between carbonyl, pyridinium nitrogen of Dexmethylphenidate**

**and the stationary phase carbamate via hydrogen bonding, and (C)**

**between the aromatic ring of Dexmethylphenidate and alkyl chain of the**

**C_18_ stationary phase via a hydrophobic H-arene interaction**

**
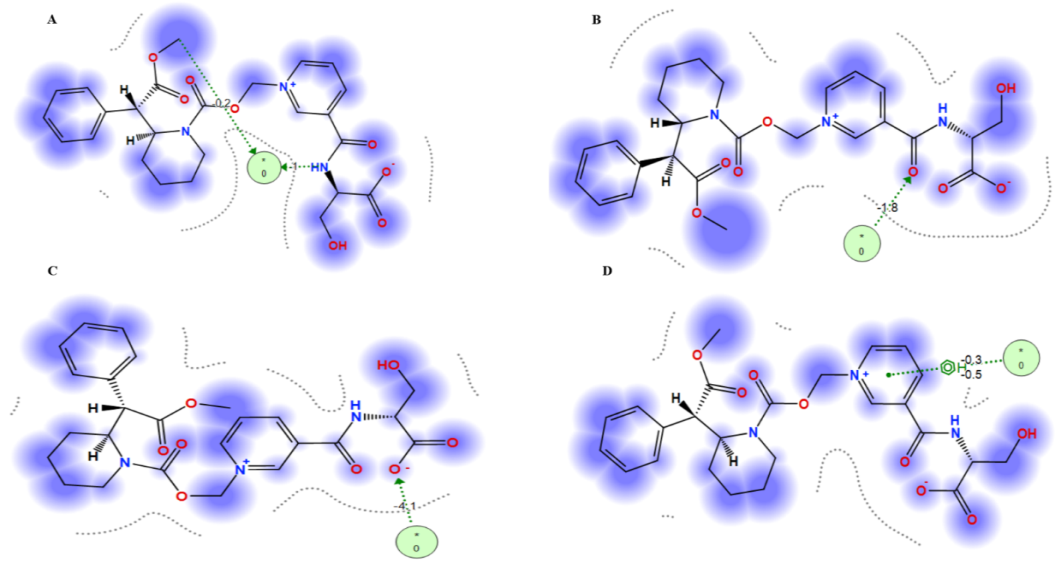
**

**Fig. S18. 2D Interaction plot showing binding interactions and energies**

**(A-C) between the carboxyl and amino groups of the amide moiety and terminal**

**carboxylic of Serdexmethylphenidate with the stationary phase carbamate groups**

**via hydrogen bonding and (D) between the aromatic ring of Serdexmethylphenidate**

**and alkyl chain of the C_18_ stationary phase via a hydrophobic H-arene interaction**

**
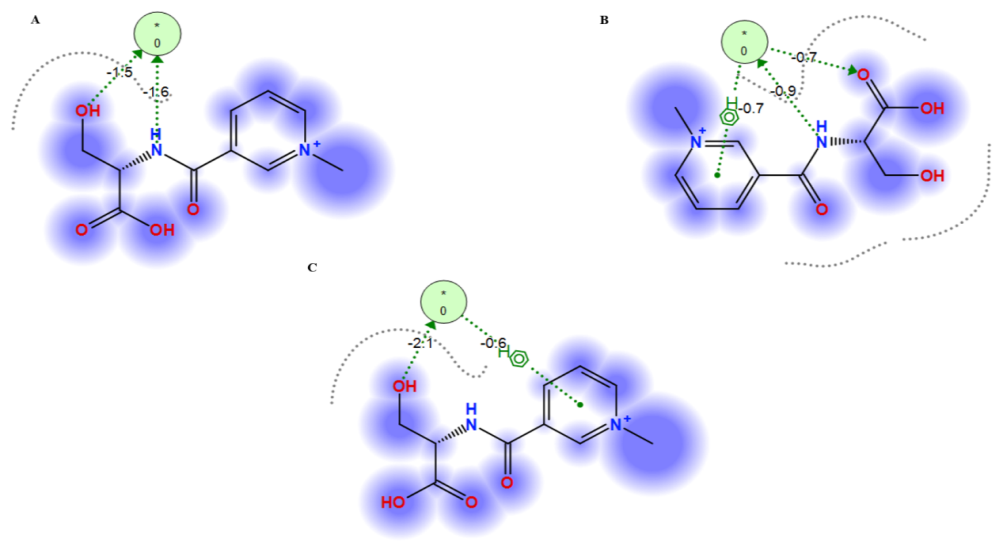
**

**Fig. S19. (A-C) 2D Interaction plot showing binding interactions and energies**

**between degradate 2, and stationary phase via hydrogen bonding and**

**hydrophobic arene interactions**

**
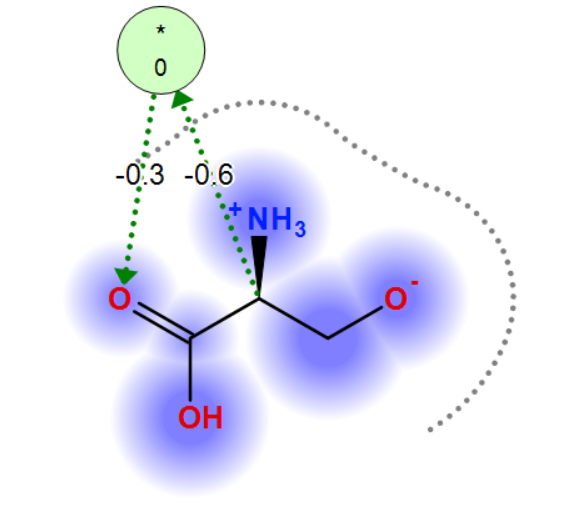
**

**Fig. S20. 2D Interaction plot showing binding interactions and energies between**

**L-serine and the stationary phase carbamate groups via hydrogen bonding**

**
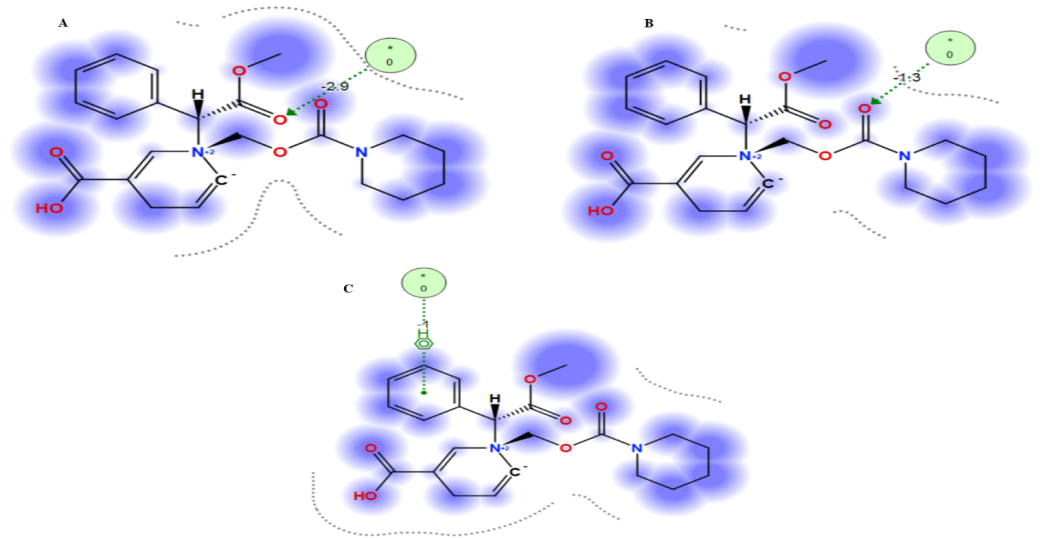
**

**Fig. S21. (A-C) 2D Interaction plot showing binding interactions and energies**

**between basic degradate, and stationary phase via hydrogen bonding**

**and hydrophobic arene interactions**

**
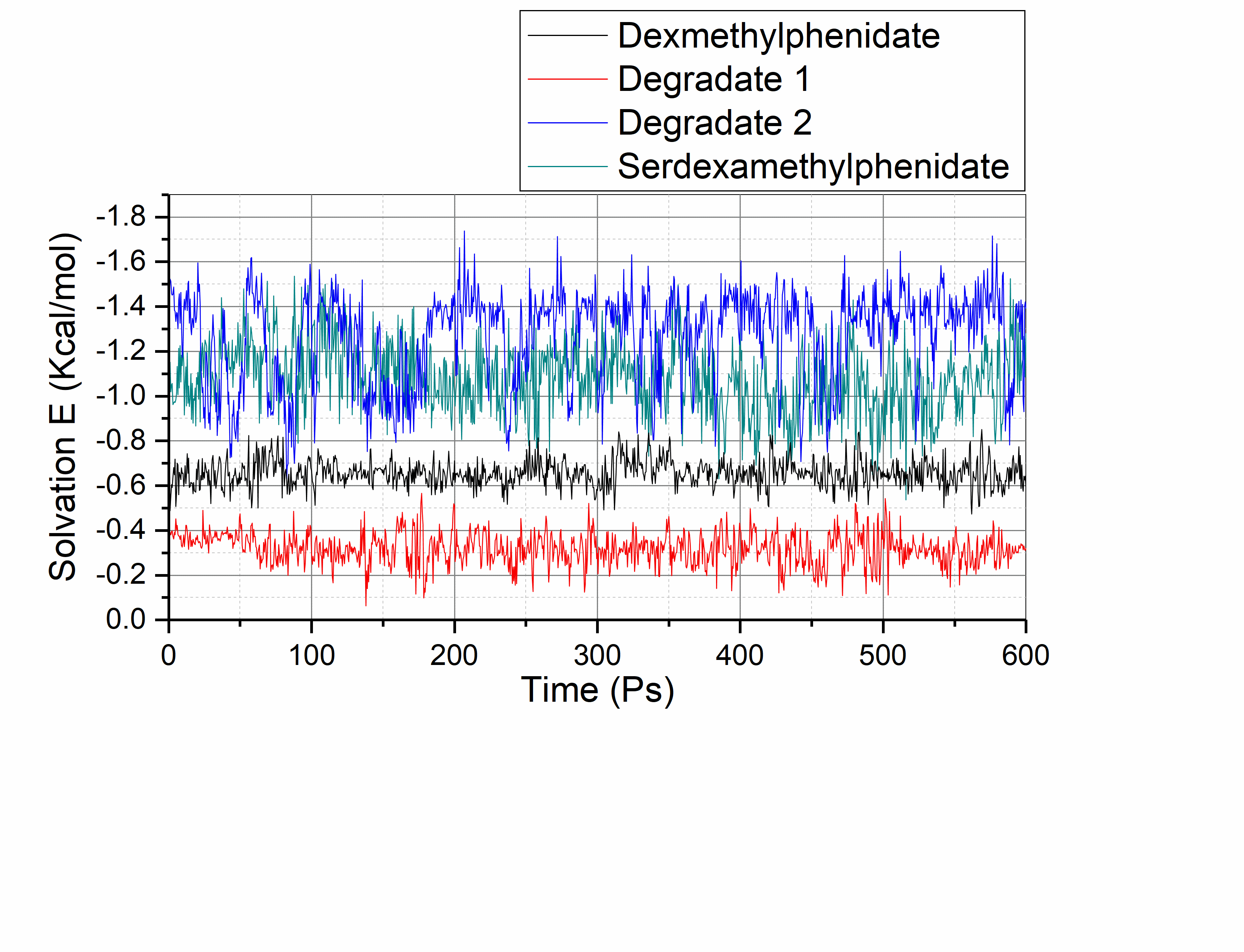
**

**Fig. S22. A calibration plot for each component in the acidic degradation**

**showing the simulation time versus its solvation energy**

**
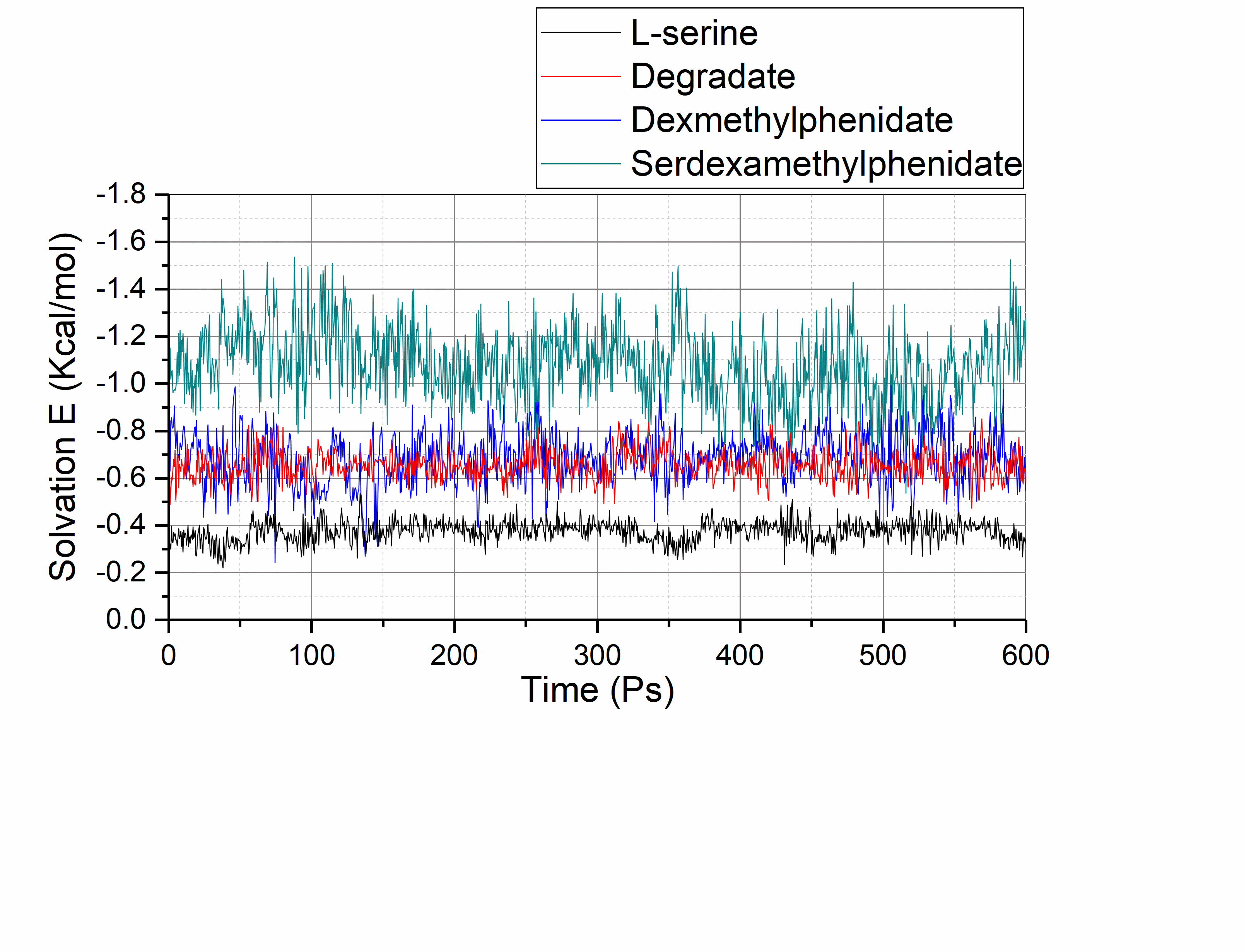
**

**Fig. S23. A calibration plot for each component in the basic degradation showing the simulation time versus its solvation energy**
